# Brittle Culm1, a COBRA-Like Protein, Functions in Cellulose Assembly through Binding Cellulose Microfibrils

**DOI:** 10.1371/journal.pgen.1003704

**Published:** 2013-08-22

**Authors:** Lifeng Liu, Keke Shang-Guan, Baocai Zhang, Xiangling Liu, Meixian Yan, Lanjun Zhang, Yanyun Shi, Mu Zhang, Qian Qian, Jiayang Li, Yihua Zhou

**Affiliations:** 1 State Key Laboratory of Plant Genomics and National Center for Plant Gene Research, Institute of Genetics and Developmental Biology, Chinese Academy of Sciences, Beijing, China; 2 State Key Laboratory of Rice Biology, China National Rice Research Institute, Chinese Academy of Agricultural Sciences, Hangzhou, China; Peking University, China

## Abstract

Cellulose represents the most abundant biopolymer in nature and has great economic importance. Cellulose chains pack laterally into crystalline forms, stacking into a complicated crystallographic structure. However, the mechanism of cellulose crystallization is poorly understood. Here, via functional characterization, we report that Brittle Culm1 (BC1), a COBRA-like protein in rice, modifies cellulose crystallinity. BC1 was demonstrated to be a glycosylphosphatidylinositol (GPI) anchored protein and can be released into cell walls by removal of the GPI anchor. BC1 possesses a carbohydrate-binding module (CBM) at its N-terminus. In vitro binding assays showed that this CBM interacts specifically with crystalline cellulose, and several aromatic residues in this domain are essential for binding. It was further demonstrated that cell wall-localized BC1 via the CBM and GPI anchor is one functional form of BC1. X-ray diffraction (XRD) assays revealed that mutations in *BC1* and knockdown of *BC1* expression decrease the crystallite width of cellulose; overexpression of *BC1* and the CBM-mutated *BC1*s caused varied crystallinity with results that were consistent with the in vitro binding assay. Moreover, interaction between the CBM and cellulose microfibrils was largely repressed when the cell wall residues were pre-stained with two cellulose dyes. Treating wild-type and *bc1* seedlings with the dyes resulted in insensitive root growth responses in *bc1* plants. Combined with the evidence that BC1 and three secondary wall cellulose synthases (CESAs) function in different steps of cellulose production as revealed by genetic analysis, we conclude that BC1 modulates cellulose assembly by interacting with cellulose and affecting microfibril crystallinity.

## Author Summary

Cellulose is an important natural resource with great economic value. Plant cellulose packs laterally into a complicated crystallographic structure, which determines cellulose quality and commercial uses. However, the mechanism of cellulose crystallization is poorly understood. Here we report that Brittle Culm1 (BC1), a COBRA-like (COBL) protein of rice, modifies cellulose crystallinity. Although previous studies have indicated the involvement of COB and COBL proteins in cellulose biosynthesis, the underlying molecular basis for this remains elusive. We demonstrate that BC1 localizes to the cell-wall and functions in a process that is distinct from that of the three secondary wall cellulose synthases (CESAs). A carbohydrate-binding module (CBM) at the N-terminus of BC1 interacts specifically with crystalline cellulose and regulates microfibril crystallite size. We conclude that BC1 modulates cellulose structure by binding to cellulose and affecting microfibril crystallinity. These findings provide new insights into the mechanism of cellulose assembly and further our understanding of the roles of COB and COBLs in cell wall biogenesis.

## Introduction

Cellulose, a class of homogenous polymers (β-1,4-glucans), represents the most abundant component of cell walls and play fundamental roles in plant growth and development. In primary cell walls (PCWs), cellulose microfibrils are cross-linked with pectin, hemicellulose and numerous proteins to define the direction and extent of cell expansion [1]. The disruption of cellulose biosynthesis at this stage generally causes a rapid loss of growth anisotropy [2]–[5]. In secondary cell walls (SCWs), cellulose that is embedded in the matrix of hemicellulose and lignin largely determines the mechanical characteristics of the wall [6]. Cellulose deficiency in SCWs often results in collapsed xylem and inferior mechanical strength [7]–[9]. In addition to its biological and physiological importance in plants, cellulose is also unequivocally among the most important natural biopolymers known to humans because of its necessity in our daily life. The commercial value of cellulose is highly correlated with its characteristics. Therefore, unraveling the factors that control the quality and quantity of cellulose will facilitate an understanding of plant cell wall biosynthesis and enable us to genetically modify cellulose.

At the molecular level, cellulose from nearly every source is equivalent. Cellulose chains further pack laterally and arrange into microfibrils with various crystal phases depending on the source. Four types of cellulose have been defined according to the arrangements of these microfibrils: Types I and II are natural forms made by plants, bacteria or algae [10], whereas types III and IV are produced from type I cellulose using chemical treatments [11]. Inconsistent with their conformational complexity, our current knowledge regarding microfibril structure and aggregation is very limited. With the aid of spectroscopic and diffraction techniques, such as small-angle neutron scattering, wide-angle X-ray scattering and solid-state ^13^C nuclear magnetic resonance spectroscopy (NMR), the glucan chains were demonstrated to assemble into crystal forms immediately upon their production. The linear crystals (approximately 3 nm thick) were found to be composed of approximately 24 cellulose chains through examination of celery collenchyma and spruce microfibril cross sections [12]–[14]. The cellulose microfibrils are further bundled into aggregates with mean diameters of 10–20 nm via noncovalent cross-linking with each other and with other polymers [12], [13], [15]. Several lines of evidence have suggested that these microfibril aggregates might be the basic cohesive unit in PCWs and in wood [13], [16].

Cellulose microfibrils are synthesized by cellulose synthesizing complexes (CSCs) called rosettes at the plasma membrane [17]. Within a microfibril, the crystalline phase in native cellulose is heterogeneous with a highly crystalline core often surrounded by amorphous forms. The degree of cellulose crystallinity is a key factor in the determination of the physicochemical behavior of cell walls [10]. However, the means by which cellulose chains pack side by side into crystalline microfibrils is largely unknown. Breakthrough findings on the crystal structure of cellulose I_α_ (triclinic unit cell) and I_β_ (monoclinic unit cell) have revealed the existence of a hydrogen-bonding network. The cellulose microfibril is assembled through inter- and intramolecular hydrogen bonds and van der Waals forces within and between the glucan chains [18], [19]. It is possible that this process occurs via self-assembly of the cellulose I_α_ and I_β_ lattices. Additionally, the geometry of CSC particles might determine the structure of cellulose crystals because the lateral dimensions of microfibrils vary with the size of the regular arrays of complex particles [7], [14]. A recent study on Arabidopsis CESA1^A903V^ and CESA3^T921I^ missense mutants revealed a correlation between CSC geometry and microfibril crystallization, providing genetic evidence for this hypothesis [20]. However, this finding has raised an additional question: whether this process requires the involvement of other components. This question is especially intriguing in plants because the natural structure of higher plant cellulose rarely contains pure cellulose I_α_ or I_β_ forms but rather contains a mixture of ordered and disordered crystal chains [21].

Increasing evidence suggests that the crystal structure of a microfibril is regulated at different levels. Some proteins other than cellulose synthases (CESAs) have been identified as being involved in cellulose biosynthesis. KORRIGAN (KOR), a membrane-bound β-1,4-glucanase, has been found to alter the level of crystalline cellulose [22], [23]. Arabidopsis CTL1 and CTL2, putative chitinase proteins, have been reported to impact cellulose assembly [24]. The influence of KOR and CTLs on cellulose structure might be a result of their roles in the proofreading of incorrectly assembled glucan chains. Calcofluor white (Calcofluor), a fluorescent cellulose dye disrupts cellulose crystallization by hydrogen bonding to the nascent glucan chains [25]. A study on the Arabidopsis mutant *mor1-1*, which exhibits reduced microtubule polymer mass, has revealed a correlation between glucan chain crystallization and the distribution of microtubule domains during cell expansion [26]. Matrix-cellulose interactions are other factors associated with cellulose crystallization. Xyloglucans affect cellulose assembly by coating on the microfibril surfaces and between microfibrils [27]. Celluloses synthesized in vitro in the absence of matrix polysaccharides have increased crystallinity compared with those synthesized in vivo [28]. However, we are still far from truly understanding how cellulose is assembled. Determining the factors that modulate microfibril crystallinity in plants remains a major challenge.

*COBRA* (*COB*) and *COB*-like genes (*COBL*s), which encode glycosylphosphatidylinositol (GPI) anchored proteins, have been found to play roles in PCW and SCW cellulose biosynthesis. The *COB* gene was identified through characterization of the *cob* mutants, which exhibit conditional root cell expansion defects and impaired crystalline cellulose production [3]. *COB* was further demonstrated to function in anisotropic growth by orienting the deposition pattern of cellulose microfibrils [29]. *COB* and eleven *COBL*s belong to a multigene family in Arabidopsis [30]. Similar families also exist in rice, maize and other plants [8], [30], [31]. Consistent with the phenotype of *cobl4*, which exhibits defects in SCWs [32], mutations in rice *Brittle Culm1* (*BC1*) and maize *Brittle Stalk2* (*BK2*) result in reduced mechanical strength [8], [33], [34]. In addition to the effects on cell wall biosynthesis, COB members (COBs) have various impacts on plant growth including root hair development [35], [36], plant height [37], and pollen development [38]. However, the precise roles of these COBs are not yet understood.

Here, we report the in-depth genetic and biochemical characterization of BC1. We demonstrate that BC1 is localized in the cell wall and interacts with crystalline cellulose through a carbohydrate-binding module (CBM). Based on the results of genetic and biophysical analyses, we conclude that BC1 modulates cellulose crystallite size and further affects cellulose biosynthesis. Our findings provide evidence that cellulose assembly requires the participation of BC1, and this is likely to be the case for COB and other COBL proteins as well.

## Results

### BC1 Is a GPI-Anchored and *N*-Glycosylated Membrane Protein

**Figure 1:**
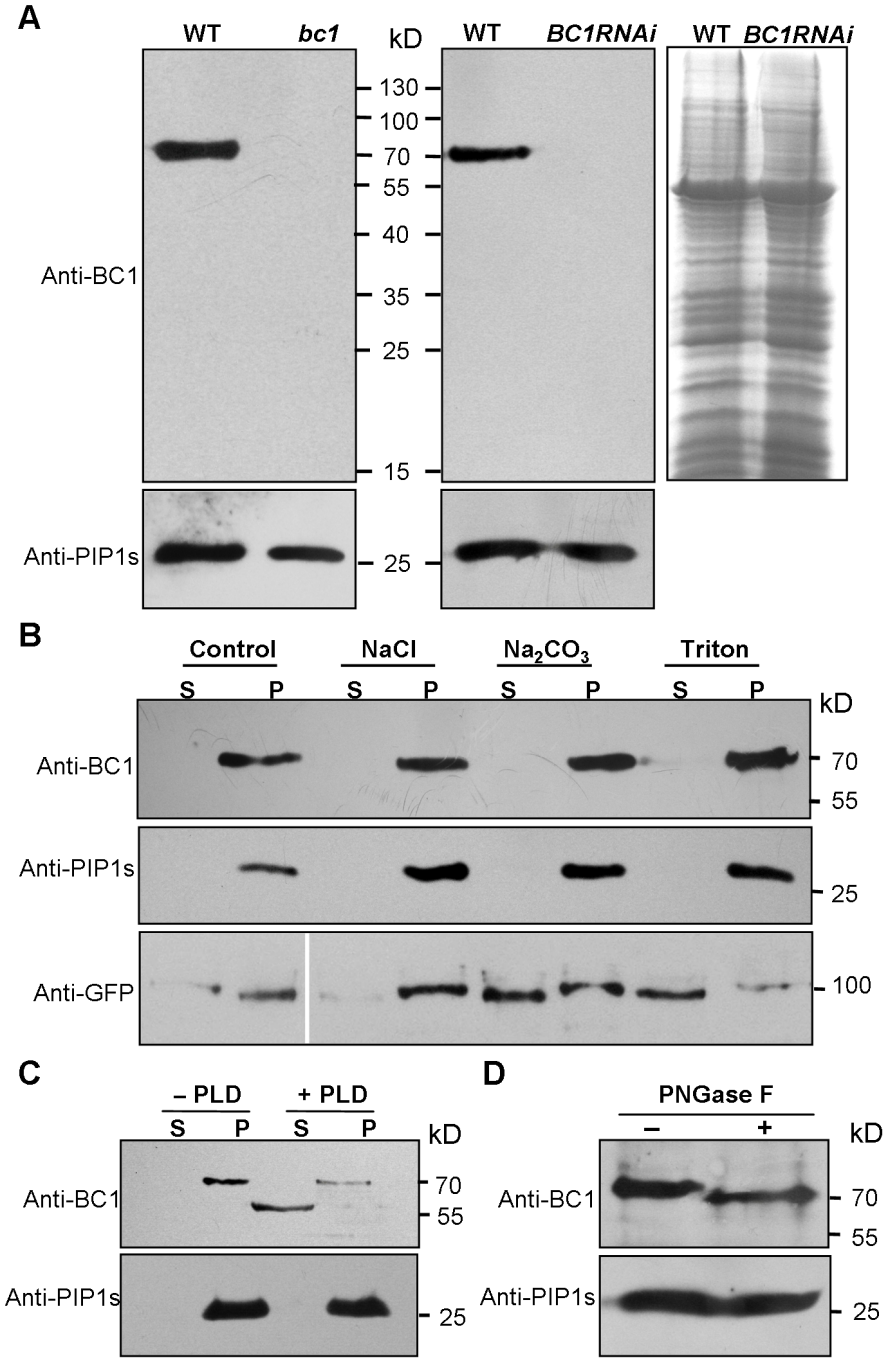
Biochemical properties of BC1. (A) SDS-PAGE gels used to separate the total membrane protein from the indicated plants were blotted and probed with anti-BC1 and anti-PIP1s antibodies. A gel stained with Coomassie blue is shown in the right panel. (B) A protein blot probed with anti-BC1 and anti-PIP1s antibodies. The treated proteins were subjected to ultracentrifugation into supernatant (S) and pellet (P) fractions. The total proteins extracted from plants expressing *BC3-GFP* were probed with anti-GFP antibody, which served as a positive control for the indicated treatments. (C) Protein blotting of buffer (−PLD)- or PLD (+PLD)-treated membrane proteins with anti-BC1 and anti-PIP1s antibodies. The proteins were subjected to ultracentrifugation into supernatant (S) and pellet (P) fractions. (D) Protein blotting of buffer (−PNGase F)- or *N*-glycosidase (+PNGase F)-treated protein extracts with anti-BC1 and anti-PIP1s antibodies. Molecular weights (kD) are indicated. PIP1s is a plasma membrane aquaporin of rice plants that facilitates the transport of water across the cell membrane, which served as loading or negative controls in these experiments.

Biochemical analysis often provides critical clues for understanding the molecular basis of a protein. To uncover the biochemical nature of BC1, we generated specific BC1 antibodies using a peptide located just after the mutation site of *bc1* as an antigen (Figure S1A and S1B). The specificity of this antibody was verified by the presence of only one band in protein extracts obtained from wild-type plants. No signal was detected from extracts obtained from *bc1* and *BC1* RNA interference (*BC1RNAi*) plants (Figure 1A) because these plants produce either prematurely terminated or few BC1 products. By protein gel blotting with this antibody, we demonstrated that BC1 is a membrane protein because it could not be solubilized by high salt (1 M NaCl), alkalinity (0.1 M Na_2_CO_3_, pH 11) or a low concentration of detergent (1% Triton X-100). Similar results were obtained with the rice plasma membrane intrinsic protein1s (PIP1s), which served as a negative control (Figure 1B). BC3 has been confirmed as a peripheral membrane protein [39]. The above treatments could extract green fluorescent protein (GFP)-tagged BC3 prepared from plants expressing the *BC3-GFP* transgene into the cytosol; thus, BC3-GFP served as a positive control (Figure 1B). Bioinformatics analysis proposed that BC1 has a hydrophobic C-terminus and a ω-cleavage site, which is a site for GPI anchor attachment [40] (Figure S1C). We therefore treated the membrane extracts with phospholipase D (PLD), an enzyme that cleaves the GPI anchor. BC1 was released into the soluble fraction and shifted faster than the membrane-bound version (Figure 1C), suggesting that it localizes to the membrane via the GPI anchor. Additionally, BC1 was found to be *N*-glycosylated because its migration in sodium dodecyl sulfate polyacrylamide gel electrophoresis (SDS-PAGE) was altered after treatment with PNGase F, an amidase that removes *N*-linked glycans from glycoproteins (Figure 1D). BC1 is therefore an authentic GPI-anchored and *N*-glycosylated membrane protein.

### BC1 Is Targeted to the Cell Wall

**Figure 2:**
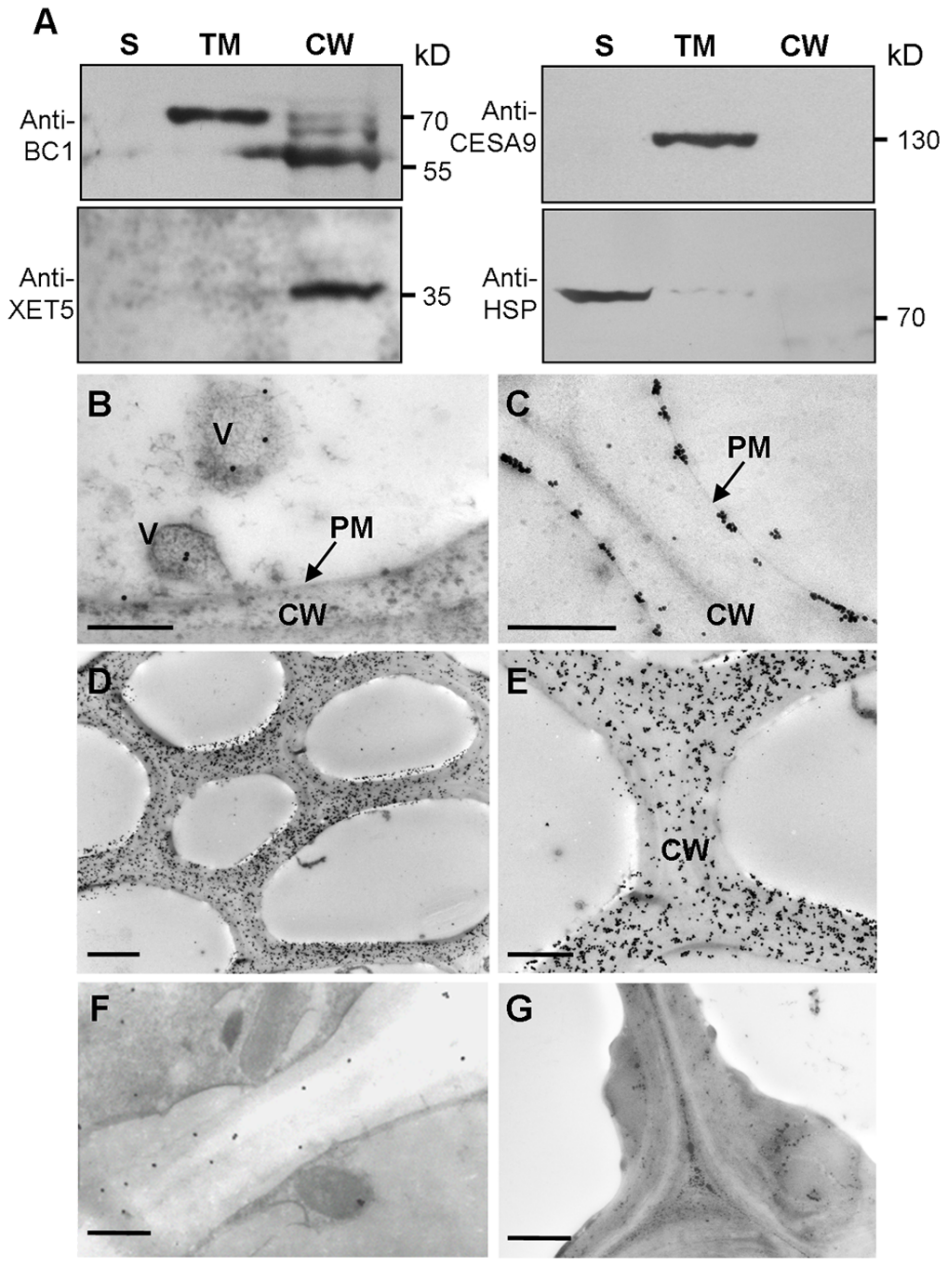
BC1 is localized in the cell wall. (A) Protein blots probed with the indicated antibodies. Antibodies against xyloglucan xyloglucosyl transferase5 (XET5), PIP1s and heat shock protein (HSP) were used for monitoring the cell wall (CW), total membrane (TM) and supernatant (S) protein fractions, respectively. Molecular weights (kD) are indicated at the right. (B–G) Immunogold labeling of BC1 in cells with thin walls (B and C) and thickening cell walls of wild-type (D and E), *bc1* mutant (F) and *BC1RNAi* (G) plants. Bars = 0.5 µm in B and C, 5 µm in D and 2 µm in E–G.

Typical GPI-modified proteins follow a secretion pathway, and ultimately reach the site at which they function. To determine BC1 localization, we performed protein gel blotting with BC1-specific antibodies. BC1 signals were observed in the total membrane and cell wall fractions (Figure 2A). Additionally, the signals detected in the cell wall fraction were the same size as those observed after PLD treatment (Figure 1C), indicating that the BC1 localized in the cell wall has lost its GPI modification. Immunogold staining was then performed to determine the distribution of BC1 at the subcellular level. In cells with thickening secondary walls, BC1 proteins were abundantly detected in the cell walls, whilst in cells with thin cell walls, BC1 proteins were found in vesicles and at the plasma membrane and cell wall (Figure 2B–2E). Few BC1 signals were detected in the secondary walls of the *bc1* mutant and *BC1RNAi* plants (Figure 2F and 2G), which served as negative controls.

Taken together, these data indicate that BC1 associates with the membrane through a GPI linkage and is released to the cell wall via the removal of this anchor.

### The CBM of BC1 Binds Crystalline Cellulose

**Figure 3:**
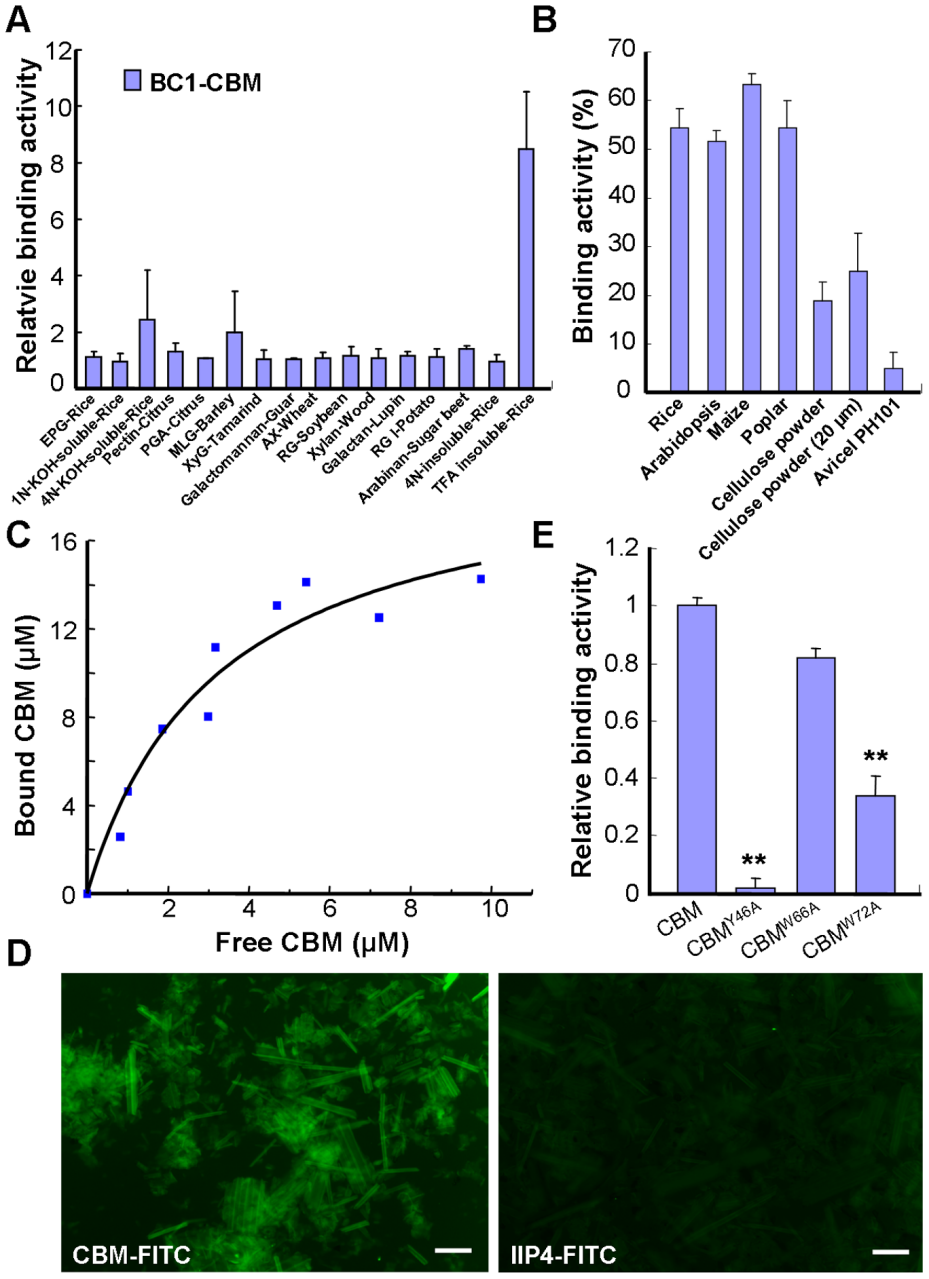
The CBM of BC1 binds crystalline cellulose. (A) Relative binding affinity of the CBM for various cell wall substrates. The relative absorbance is derived from the protein blot signals shown in Figure S3. The data are presented as the mean ± SE (*n*≥2). (B) Binding affinity of the CBM for crystalline cellulose from plants and commercial products determined via ELISA. The data are presented as the mean ± SE (*n* = 3). (C) Dissociation constant value for CBM binding to rice crystalline cellulose obtained from a representative experiment. (D) Immunochemical staining of rice crystalline cellulose with recombinant CBM or IIP4 (a negative control) using anti-His and anti-FITC as the primary and secondary antibodies, respectively. Bar = 100 µm. (E) Relative binding affinity of the wild-type and point-mutated CBMs to commercial cellulose (20 µm powder) determined via ELISA. The data are presented as the mean ± SE (*n*≥2, **P<0.01 by Student's *t*-test).

Next, we wanted to investigate how BC1 associates with the cell wall. Pfam searching revealed that BC1 has a putative CBM at its N-terminus (Figure S2A). This sequence shows low similarity to CBM2a and CBM2b, which bind to cellulose and xylan, respectively, and were found in bacteria and fungi (Figure S2B). To determine whether the putative CBM domain can bind carbohydrate polymers, we purified a His-tagged recombinant protein containing this domain in *E. coli* and analyzed its binding activity with various polysaccharide polymers (Figure S3). Carbohydrate microarray and the quantification of binding activity to carbohydrates showed that this CBM specifically bound rice-derived crystalline cellulose (Figures S3 and 3A). To examine whether this CBM binds cellulose from other sources, its binding affinity for different celluloses was further examined by enzyme-linked immunosorbent assay (ELISA). This CBM showed comparable binding affinity for crystalline cellulose from several plant species and had varied affinity for some commercial cellulose products (Figure 3B). The dissociation constant value for CBM binding to rice residues that were insoluble in Updegraff reagent and rich in crystalline cellulose was 4.2 µM based on a representative experiment (Figure 3C), and the mean equilibrium dissociation constant (*K*_d_) from three independent replicates was 4.3±0.7 µM. To visualize the interaction between the CBM and cellulose, the recombinant CBM was incubated with rice crystalline cellulose. The cellulose microfibrils became fluorescent after immunostaining the CBM with an anti-His antibody, whereas no signal was detected after staining with an antibody against a known nuclear protein IIP4 [41] (Figure 3D). Therefore, the CBM of BC1 shows preferential affinity for crystalline cellulose.

### The CBM Is a Functional Domain

It has been reported that some aromatic amino acids in bacterial and fungal CBMs are critical for binding [42]. The CBM of BC1 also possesses such conserved residues (Figures S2A and S4A). To investigate the necessity of these amino acids for cellulose binding, we chose three aromatic residues that are highly conserved among members of the COB family in Arabidopsis and rice for the generation of point-mutated recombinant CBMs in *E. coli* (Figure S4). In vitro binding assays showed that CBM^Y46A^ and CBM^W72A^ exhibited significantly reduced binding ability to commercial crystalline cellulose, whereas CBM^W66A^ retained a binding ability similar to that of the wild type (Figure 3E). These results indicated that certain aromatic residues contribute to the interaction between this CBM and crystalline cellulose.

**Figure 4:**
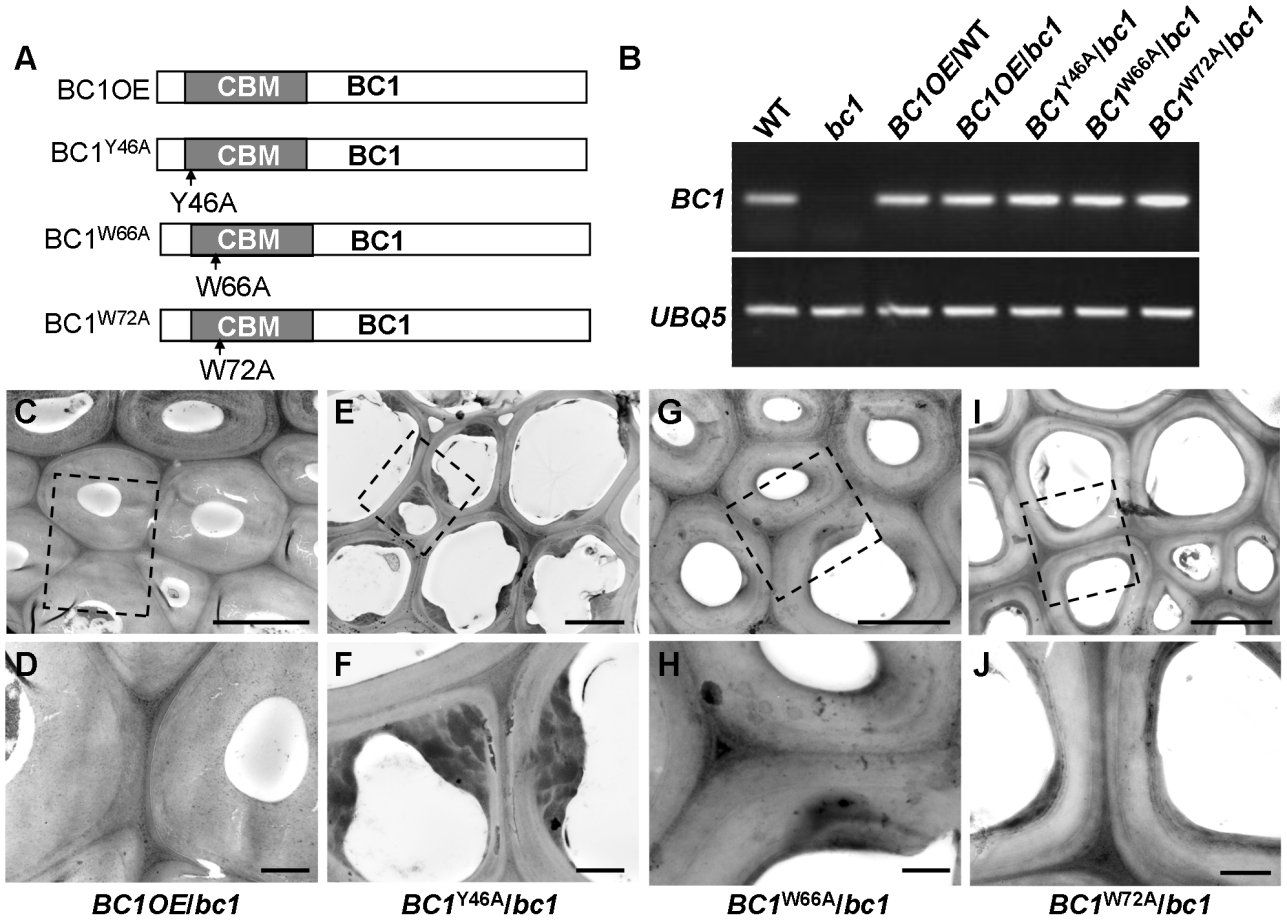
The CBM in BC1 is a functional domain. (A) BC1 and BC1 variants used for the transformation assay. (B) RT-PCR analysis, confirming that the transgenic plants with *bc1* (/*bc1*) and wild-type (/WT) backgrounds have increased *BC1* expression levels. (C–J) TEM micrographs of sclerenchyma cell cross-sections from plants overexpressing the wild-type *BC1* (C and D) and CBM-mutated *BC*, including *BC1* ^Y46A^ (E and F), *BC1* ^W66A^ (G and H), and *BC1* ^W72A^ (I and J). The dashed rectangles indicate the parts being magnified in the lower panels (D, F, H, and J). Bars = 5 µm (C, E, G, and I) and 1 µm (D, F, H, and J).

Our previous work showed that *bc1* mutant plants have a reduced cellulose level [8]. Here, transmission electron microscopy (TEM) further revealed that the SCW of *bc1* is significantly thinner and abnormal due to the accumulation of electron-dense stained materials, whereas the wild-type secondary walls are evenly thickened with three layers (Figure S5). To investigate the functional importance of the CBM in BC1, we overexpressed wild-type and CBM-mutated *BC1* variants in a *bc1* mutant background (Figure 4). The cellulose content and SCW structure were examined to determine whether these constructs could rescue the *bc1* mutant phenotype. As shown in Table 1 and Figure 4, the plants expressing *BC1* and *BC1* ^W66A^ had wild type-like cellulose content and wall structure, whereas plants expressing *BC1* ^W72A^ showed partial recovery of both phenotypes. However, the plants expressing *BC1* ^Y46A^ had *bc1*-like phenotypes. These results suggest that the CBM is important for the function of BC1.

**Table 1:** BC1 affects cellulose crystallite size and cellulose content.

Genotype	WT	*bc1*	*BC1* ^Y46A^/*bc1* 4	*BC1* ^W66A^/*bc1* 4	*BC1* ^W72A^/*bc1* 4	*BC1OE*/*bc1* 4	*BC1OE*/WT^5^	*BC1RNAi* 5
Cellulose^1^	397.7±6.2	336.8±3.0*	299.7±5.6*	403.5±4.7	372.9±7.1*	395.2±7.6	419.4±9.0*	226.2±8.6*
RCI^2^	59.9±0.9	44.5±0.8*	37.0±0.5*	54.6±0.1	50.9±0.6*	57.4±0.3	64.3±0.7*	20.2±1.5*
Crystallite size^3^	20.3±0.6	16.3±0.3*	15.7±0.1*	19.8±0.5	16.3±0.5*	20.3±0.3	19.5±0.1	10.3±0.4*

Cell wall residues were prepared from the 3^rd^ internodes from 4-month old wild type, *bc1*, and indicated transgenic plants.

1 The cellulose content is determined by Updegraff quantification after TFA hydrolysis and is presented as µg cellulose per mg alcohol-insoluble residues (AIRs), mean value ± SE. Asterisks indicate *P*<0.01 Student's *t* test with respect to wild type, *n* = 4.

2 As determined by Segal method [47] and presented as percentage of crystalline in cell wall components, mean value ± SE. Asterisks indicate *P*<0.01 Student's *t* test with respect to wild type, *n* = 3.

3 As determined by synchrotron X-ray analysis and presented as Å ± SE. Asterisks indicate *P*<0.01 Student's *t* test with respect to wild type, *n* = 3.

4, 5 Expressing the indicated constructs in *bc1* mutant and wild-type plants, respectively.

### Binding of BC1 to Cell Walls Depends on the CBM and GPI Modification

**Figure 5:**
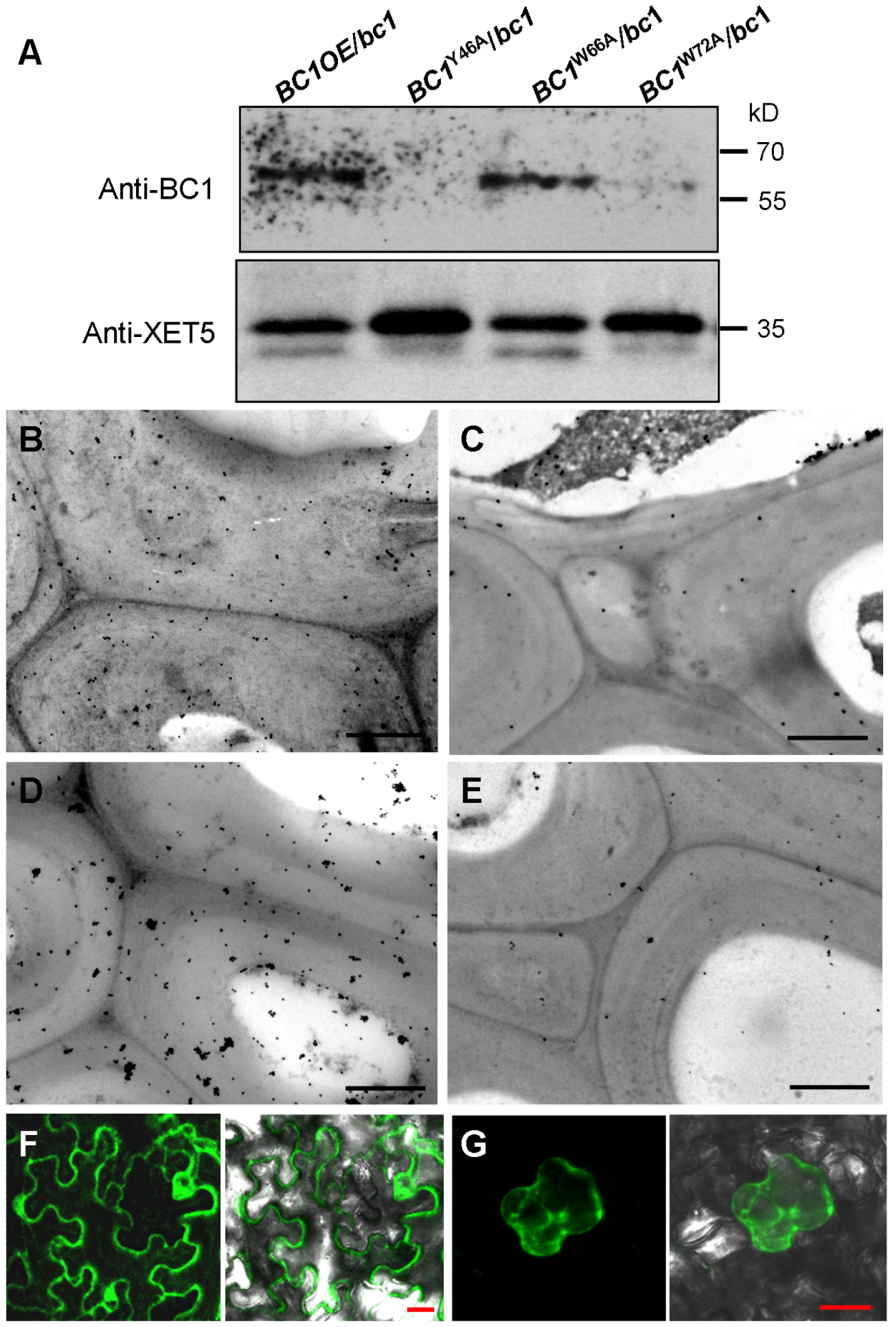
The CBM and GPI-substitution are essential for the cell wall localization of BC1. (A) Protein blotting of BC1 in cell wall fractions that were extracted from plants expressing wild-type *BC1*, *BC1* ^Y46A^, *BC1* ^W66A^ and *BC1* ^W72A^. XET5 served as a loading control. (B–E) Immunogold labeling of BC1 in the secondary walls of mature plants expressing wild-type *BC1* (B), *BC1* ^Y46A^ (C), *BC1* ^W66A^ (D) and *BC1* ^W72A^ (E). (F) Transient expression of GFP-CBM in tobacco leaf epidermal cells. (G) Mannitol-induced plasmolysis to examine GFP-CBM in tobacco epidermal cells. Bars = 1 µm in B–E and 20 µm in F and G.

Given that cell wall-localized BC1 represents one functional form of this protein, we wanted to determine which domain contributes to this localization, and the CBM is one potential candidate. We therefore incubated the purified wild-type and point-mutated CBMs with cross-sections of rice internodes. Immunogold labeling analysis revealed that CBM^Y46A^ and CBM^W72A^ proteins exhibited reduced affinity for cell walls compared with the wild-type and CBM^W66A^ proteins (Figure S6). We further utilized the transgenic plants expressing wild-type and CBM-mutated *BC1* to examine BC1 abundance on cell walls. Protein gel blotting with BC1 antibodies revealed comparable BC1 levels in cell wall extracts from plants expressing wild-type *BC1* and *BC1* ^W66A^; however, low BC1 signals were noted in cell wall extracts from plants expressing *BC1* ^Y46A^ and *BC1* ^W72A^ (Figure 5A). Immunogold labeling also showed reduced BC1 signals in the cell walls of plants expressing *BC1* ^Y46A^ and *BC1* ^W72A^, compared with the plants expressing wild-type *BC1* and *BC1* ^W66A^ (Figure 5B–5E). The CBM is therefore essential for the cell-wall localization of BC1.

To examine whether the CBM alone can be targeted to the cell wall in vivo, we transiently expressed a construct harboring GFP-CBM in tobacco leaves. GFP signals were observed in the plasma membrane and cytoplasm (Figure 5F). Mannitol-induced plasmolysis clearly revealed an absence of GFP signals on the cell wall (Figure 5G). Thus, we hypothesized that an additional domain might be essential for the localization of BC1 on the cell wall. Previous works have revealed that the GPI modification of COB is important for its delivery [29]. We therefore replaced the C-terminus of BC1 with GFP at the ω-cleavage site and expressed the resulting construct in *bc1* mutant plants. The BC1^Δω^GFP signals retained inside cells due to the lack of a GPI anchor as revealed by PLD treatment and in vivo examination of GFP signals in the root cells of transgenic plants (Figure S7A and S7B). The cellulose level of transgenic plants remained *bc1*-like (Figure S7C). Combined with the results shown in Figure 2, without the GPI substitution, BC1 proteins lack the PLD cleavage site and are unable to be delivered and released to the apoplast.

Therefore, the CBM domain and the GPI modification contribute together to the cell wall localization of BC1.

### BC1 Affects Cellulose Biosynthesis through a Process That Is Distinct from That of the CESAs

BC1 is critical for secondary wall cellulose biosynthesis [8]. To further dissect the role of BC1 in cellulose production, we generated *BC1* knockdown plants (*BC1RNAi*). An examination of cell wall composition showed that the cellulose content of *BC1RNAi* plants was significantly decreased (Table 1). Noncellulosic sugar content in *bc1* and *BC1RNAi* plants was increased (Table S1), which is similar to the alterations noted in rice *cesa* mutants [43], [44]. The increase in noncellulosic sugars might be a feedback response to the cellulose deficiency. Therefore, similar to the CESAs, BC1 is required for cellulose biosynthesis.

**Figure 6:**
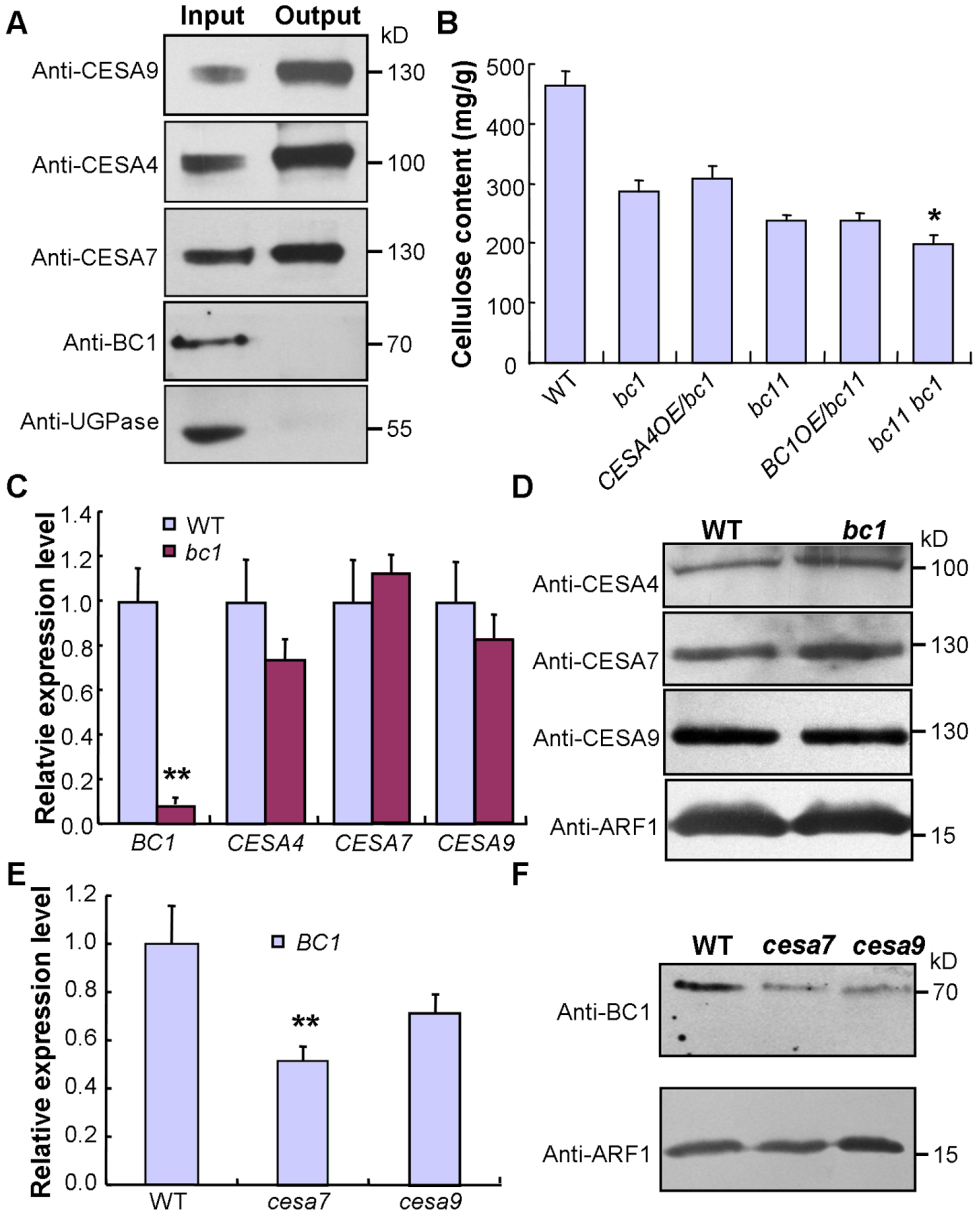
BC1 and SCW CESAs function in different steps of cellulose production. (A) A Co-IP assay of BC1 and three SCW CESAs in transgenic rice plants expressing the *FLAG-CESA9* transgene. Proteins before (input) and after (output) immunoprecipitation were probed with the antibodies as indicated. UGPase is a cytoplasmic protein for synthesis of sucrose, which served as a negative control. (B) Cellulose content of the 2^nd^ internodes of 5-month old wild type, *bc1*, *bc11* and *bc1 bc11* mutants and plants expressing *BC1* or *CESA4* transgenes. The data are presented as the mean ± SE (*n* = 3, *P<0.05 by Student's *t*-test with respect to *bc11*). (C and D) Expression levels of *BC1* and three *CESA*s in wild-type and *bc1* plants as examined by qRT-PCR (C) and protein blotting (D). The data are presented as the mean ± SE (*n* = 3, **P<0.01 by Student's *t*-test). (E and F) Expression levels of *BC1* in wild type and *cesa* mutants as examined by qRT-PCR (E) and protein blotting (F). The data are presented as the mean ± SE (*n* = 3, **P<0.01 by Student's *t*-test). ADP ribosylation factor1 (ARF1) is a small GTPase implicated in vesicle trafficking, which served as a loading control in (D and F). Molecular weights (kD) are indicated at the right.

This conclusion was further supported by data from the co-expression assay. An examination of the co-expression network revealed that *BC1* is highly co-expressed with three *CESA* genes involved in SCW cellulose formation, and these four genes have consistent spatiotemporal expression profiles based on available microarray data (Figure S8). Therefore, an investigation of the relationship between BC1 and the three SCW CESAs will provide clues for understanding BC1 function. The co-expression phenotype first promoted us to examine whether BC1 is a component of the CSC complex. To address this possibility, we generated transgenic plants that harbored a FLAG-tagged *CESA9* transgene and subsequently purified FLAG-CESA9 and the associated proteins by co-immunoprecipitation (Co-IP). Using SCW CESA- and BC1-specific antibodies, three SCW CESA proteins were precipitated, whereas we failed to detect BC1 and the negative control protein, UDP-glucose pyrophosphorylase (UGPase) (Figure 6A). Although this experiment could not absolutely rule out the possibility that BC1 is present in the CSC complex, when combined with the evidence that BC1 functions in the cell wall, BC1 is not likely to be a component of the CSC complex formed by the three SCW CESAs. Next, we questioned whether BC1 and SCW CESAs are genetically associated. Because *bc1* and *bc11*, a previously reported *cesa4* mutant [43], have the same genetic background, we produced a *bc1 bc11* double mutant and overexpressed *BC1* or *CESA4* in each of the single mutant. Neither *BC1* nor *CESA4* overexpression was able to recover the cellulose level in *bc11* or *bc1*, indicating that these proteins have distinct biochemical activities. Additionally, the cellulose content in the *bc1 bc11* double mutant was lower than that in either single mutant (*bc11* or *bc1*) (Figure 6B). We further analyzed the expression of these genes in the relevant mutants. In *bc1* plants, the RNA and protein levels of the three SCW CESAs were comparable to that of the wild type (Figure 6C and 6D). However, the RNA and protein levels of *BC1* were obviously downregulated in *bc1* and the *cesa* mutants (Figure 6C, 6E, and 6F). Taken together, these results indicate that both BC1 and CESAs are required for cellulose production but may function in different steps. The process mediated by BC1 occurs after (or is coupled with) glucan chain polymerization, which is catalyzed by CESAs.

### BC1 Modifies Cellulose Structure

Cell wall-localized proteins are generally involved in cell wall assembly or remodeling [17]. Considering that one functional form of BC1 is in the cell wall, an investigation of its impact on cellulose crystallinity would be reasonable. We therefore measured the relative crystallinity index (RCI) of intact wall residues from wild type, *bc1* and the CBM point-mutated *BC1* transgenic plants with X-ray diffraction (XRD). RCI roughly reflects the proportion of crystalline to amorphous cellulose [45], [46]. According to the generated diffractograms (Figure S9A and S9B), use of the Segal method [47] revealed significantly reduced RCI values in the *bc1* and *BC1RNAi* plants. The RCI was almost restored to the wild-type level by expression of *BC1* ^W66A^ and wild-type *BC1* in *bc1*. Plants expressing *BC1* ^Y46A^ had a lower RCI than *bc1* plants; however, plants expressing *BC1* ^W72A^ had an RCI value between that of wild type and that of *bc1* (Table 1). More interestingly, when *BC1* was overexpressed in the wild-type background (*BC1OE*/WT), the RCI was increased more than when *BC1* was overexpressed in *bc1* mutants (*BC1OE/bc1*) (Table 1). Furthermore, high-energy synchrotron radiation XRD was employed to obtain the crystallite size for a more accurate estimation of microfibril structure. Synchrotron XRD data generated by the Scherrer equation [48] indicated a reduction in the crystallite width in *bc1* and CBM-mutated (*BC1* ^W72A^ and *BC1* ^Y46A^) transgenic plants. The crystallite size of *BC1RNAi* plants was decreased by approximately 50% compared with wild type (Table 1). Although overexpressing *BC1* ^W66A^ and wild-type *BC1* in *bc1* rescued the crystallite size to the wild-type level, increasing the amount of *BC1* transcripts could not increase the crystallite width beyond that of the wild type.

**Figure 7:**
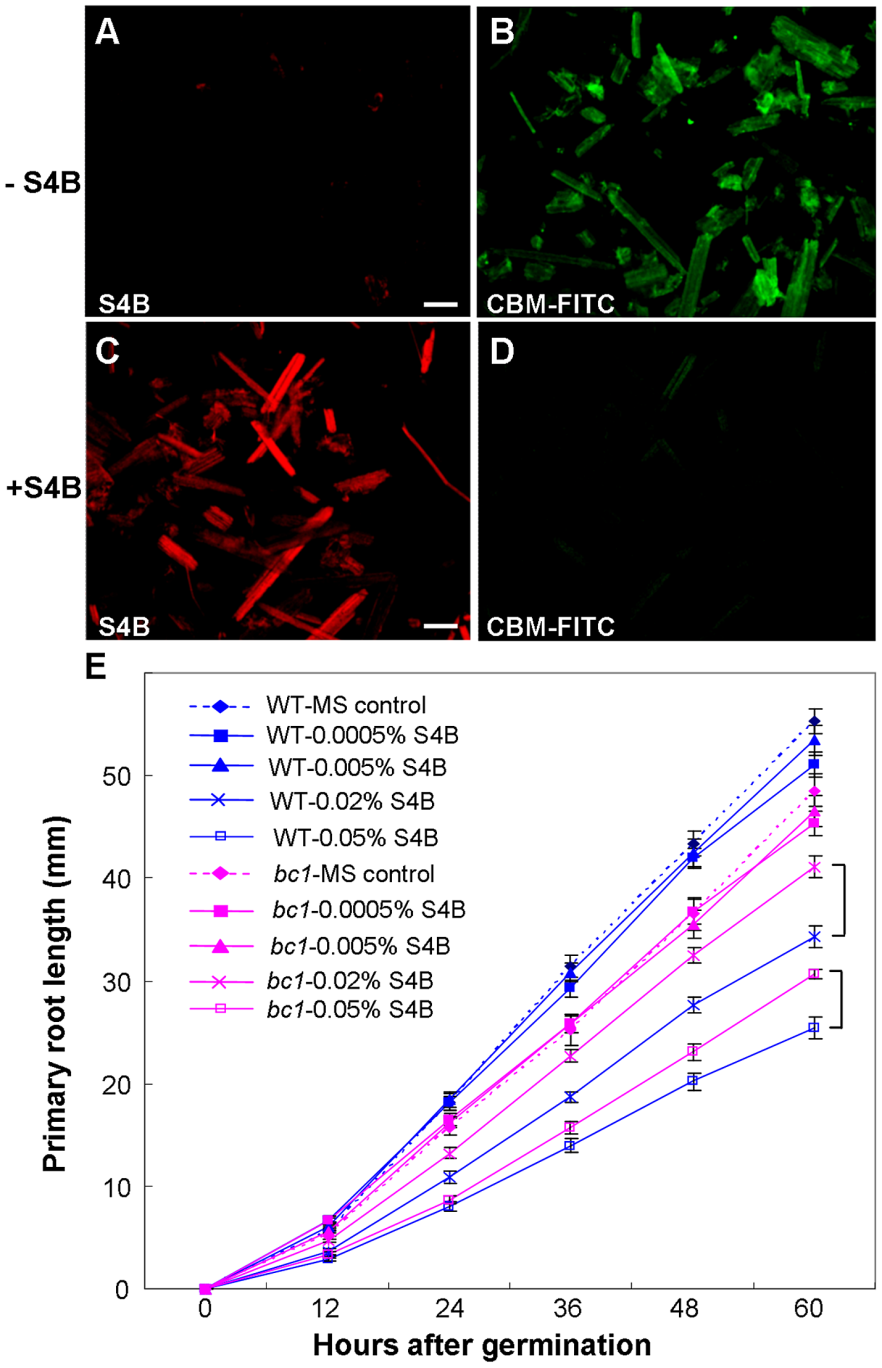
BC1 modulates cellulose crystallization. (A–D) Immunostaining of CBM with unstained or pre-stained rice crystalline cellulose (0.01% S4B, w/v) using anti-His and anti-FITC as primary and secondary antibodies, respectively. Bar = 100 µm. (E) Effects of S4B on root growth in wild-type and *bc1* seedlings. Primary root length was measured at the indicated time. MS, Murashige and Skoog medium. The data are presented as the mean ± SE (*n*≥15). Square brackets indicate the varied responses to one concentration of S4B between wild-type and *bc1* seedlings.

Pontamine fast scarlet 4B (S4B) and Calcofluor are fluorescent cellulose dyes that influence microfibril crystallization [16], [25], [49]. To determine the means by which BC1 interacts with cellulose, we incubated the recombinant CBM of BC1 with rice cellulose residues that were pre-stained with S4B or Calcofluor. The CBM failed to label the cellulose microfibrils after staining with the dyes (Figures 7A–7D and S10), indicating that BC1 might have a similar mechanism of interaction with cellulose. We further treated the wild-type and *bc1* seedlings with media containing various amounts of S4B or Calcofluor and measured the root lengths. High levels of S4B repressed the root growth of the wild-type plants, whereas Calcofluor promoted root growth at low concentrations and inhibited root growth at high concentrations (Figure S9C and S9D). However, *bc1* root growth was consistently insensitive to treatments with both dyes, with the exception of a strong inhibition noted at a high concentration (Figures 7E and S10E). The dissimilar responses to these fluorescent dyes imply that *bc1* has a cellulose crystallinity status that is distinct from that of the wild type.

## Discussion

Cellulose is the one of most abundant biopolymers on earth. Understanding the factors that control cellulose biosynthesis will allow us to manipulate its quantity and quality and improve its bioconversion efficiency when using it as a source for biofuel production. However, cellulose biosynthesis involves complicated processes, such as glucan chain polymerization, packaging, aggregation into larger microfibrils, and deposition. Thus, many components are likely to be involved in cellulose production, and COBs are one such essential component.

### Understanding BC1 Function Based on Its Structure

The COB family is widespread in plant genomes [31]. Although COB and COBL members play roles in PCW and SCW cellulose biosynthesis [3], [8], [30], [34], [37], the molecular basis is unknown. To understand the functions of COB and COBL proteins, an examination of their domain structure is necessary. COBs share three common domains: the N-terminal domain for binding carbohydrates, the Cys-rich CCVS domain and the hydrophobic C-terminal domain for attaching a GPI moiety [30], [31]. BC1 is predicted to possess all of these conserved domains. Here, we have demonstrated that the N-terminal CBM domain preferentially interacts with crystalline cellulose. The interaction noted in type A CBM occurs via aromatic residues, which facilitate the formation of hydrogen bonds and van der Waals contacts between essential groups and the cellulose surface [42], [50], [51]. In this work, we investigated the biochemical nature of the CBM and examined the contribution of certain aromatic residues by mutagenesis. An in vitro binding assay and in vivo rescue analysis revealed that Y46 and W72 are critical residues for binding with cellulose and targeting to cell walls. The importance of this domain and these residues was highlighted in the previous work [52]. Moreover, the mutated residues in Arabidopsis *cob-1* to *cob-3* are located in this domain and an aromatic residue (W55) is mutated in *cob-3* [30]. Another functional domain of BC1 is the C-terminus, which is where the GPI moiety is attached. GPI anchor is a type of posttranslational modification that facilitates the delivery of certain proteins to the cell wall. We have showed that BC1 is a GPI-modified protein because PLD treatment successfully removed this anchor and solubilized BC1. Cell wall-localized BC1 is the same size as BC1 after PLD cleavage. Without GPI substitution, BC1 (as well as the CBM alone) cannot be released to the cell wall and recover the *bc1* phenotype. However, *bc1* plants expressing *BC1*s that retain the GPI anchor but contain CBM variants (*BC1* ^Y46A^ and *BC1* ^W72A^) failed to recover cellulose levels to the wild type and showed reduced BC1 abundance in the cell wall based on protein blotting assays and immunogold labeling. Our data suggested that GPI modification and the CBM are critical for BC1 delivery and binding to cellulose. Deficiency in either domain does not allow BC1 targeting to the cell wall. The CCVS domain is the third conserved domain in COBs and was proposed to be involved in disulfide bond formation or metal ion binding due to the recover of a yeast mutant deficient in a phytochelatin synthase [30], [53]. Further studies are needed to uncover the exact biochemical function of the CCVS domain.

### BC1 Is Required for Cellulose Assembly

As a cell wall-localized protein and required for secondary wall cellulose biosynthesis, BC1 is very likely to function in cellulose assembly. In fact, the question of how plants arrange their native cellulose structure remains unanswered. Little is known about the mechanism by which glucan chains stack and the identities of proteins that modulate cellulose crystallinity in plants.

Cellulose chains are arranged in parallel to form a mixed higher structure of ordered (crystals) and disordered (amorphous cellulose) forms [12], [13], [18], [19]. Therefore, a protein implicated in cellulose assembly must be localized to the cell wall and must interact with cellulose. Based on biochemical and biophysical evidence, BC1 meets the above two requirements. Direct evidence for the ability of BC1 to modify cellulose crystallinity was obtained from XRD data from several transgenic plants. *bc1* plants had reduced RCI values and crystallite size. The plants expressing CBM-mutated BC1 showed varied crystallite size, which is consistent with their in vitro binding activity. Overexpressing *BC1* in the *bc1* background rescued the *bc1*-like RCI and crystallite width to the wild-type levels; overexpressing *BC1* in the wild-type background increased the RCI value to greater than that of the wild type by altering the proportion of crystalline to amorphous components. More importantly, although the amount of cellulose in plants expressing *BC1* ^W72A^ was largely recovered, the crystallite size remained similar to that of *bc1*. These data strongly suggest that BC1 modulates cellulose structure in terms of its crystallinity. The physiological responses of the *bc1* and wild-type seedlings to S4B and Calcofluor further demonstrated that *BC1* mutation alters cellulose crystallization. Two hypotheses have been proposed for how plants assemble cellulose structure [14]. In addition to the self-assembly model, the geometry of CSCs may determine cellulose crystallinity because subtle changes in CESA conformation will affect the passage and proper alignment of glucan chains into a crystalline microfibril. A recent study reported that mutations in the transmembrane helix structure of AtCESA1 and AtCESA3 induce organizational alterations in glucan chains [20]. Additionally, there is no conclusive evidence to indicate that packing cellulose into crystals requires the involvement of proteins, although several proteins, such as KOR1 and CTL1, have been reported to influence the level of crystalline cellulose [23], [24], [26]. Here, our intensive analyses of the biochemical functions of BC1 domains and impacts on cellulose crystallinity strongly suggest that BC1 interacts with crystalline cellulose and modulates cellulose structure.

The next intriguing question is how BC1 modulates cellulose crystallinity. The crystal structure of CBM will provide insights into the mechanisms of protein-carbohydrate interaction [51], [54]. Because this crystal structure is not available, it is currently difficult to clearly address this question. However, we found that the binding ability of the CBM of BC1 was largely decreased if cellulose microfibrils were pre-stained with S4B and Calcofluor, which bind cellulose via noncovalent bonds. BC1 might interact with and affect cellulose crystallization in related ways.

### The Relationship between BC1 and CESAs

Of the many processes that are required for cellulose biosynthesis in higher plants, glucan chain polymerization and crystallization are two coupled and biophysically linked processes [55]. Therefore, a deficiency in cellulose content is accompanied by aberrant crystal microfibrils, and vice versa [20], [24]. CESA proteins are known to catalyze cellulose polymerization at the plasma membrane [56], [57]. Mutations in BC1 cause reductions in crystallite size and cellulose level, similar to the alterations in *cesa* mutants [20]. Then, which response is the primary effect to the mutations of *BC1*? CESAs are used as markers to address the particular processes that BC1 might participate in. In this study, BC1 was not found in the CSC complex based on the Co-IP assay. Gene expression assay of *BC1* and *CESA* levels and compositional analysis of cellulose content in the *bc1*, *bc11* and *bc1 bc11* mutants determined that BC1 and CESAs are genetically associated. In addition to the distinct functional sites and biochemical activities of BC1 and CESAs, BC1 is likely to modulate cellulose crystallinity, mediating a distinct process from cellulose polymerization, although it also affects cellulose content. However, we do not rule out the possibility that BC1 also functions at the plasma membrane, where it may associate with CESAs.

The size and structure of microfibrils play critical roles in tensile strength and growth control. Here, we demonstrated that BC1 is a key factor in the modification of cellulose structure in SCW and the regulation of mechanical support. COBs share a similar domain structure with BC1 and exhibit organ-specific expression patterns [30]. These proteins may have related yet distinct roles. COB was identified to control anisotropic growth in root cells [29]. Although the growth of plant cells is not well understood, microfibril crystallinity was found to be correlative with the maintenance of anisotropic expansion during rapid growth [26], [58]. As a putative cellulose interacting protein, COB appears to modulate cellulose structure and content, which in turn may affect the lateral slippage ability of cellulose microfibrils and unidirectional cell growth. Therefore, this study provides new insights into the mechanisms of action of COB and COBLs. It is expected that COB and other COBL proteins may interact with cellulose or other polymers and modulate cell wall structure in different cell types.

## Materials and Methods

### Plant Materials and Genetic Analysis

The wild type, *bc1* and relevant transgenic plants used in this study were cultivated in the experimental fields at the Institute of Genetics and Developmental Biology in Beijing or Sanya (Hainan Province, China) during the natural growing seasons. The *tos17* insertion mutants in *CESA7* and *CESA9* were purchased from NIAS (rice *Tos17* insertion mutant database, http://tos.nias.affrc.go.jp/). The *bc1 bc11* double mutant was generated by crossing *bc1* with *bc11* plants [43]. The homozygous plants were isolated from F2 plants using the primers listed in Table S2.

For preparation of *BC1RNAi* plants, the specific fragment of *BC1* was amplified by the primers (Table S2) and inserted into the binary vector *pKANIBAL*. The resulting construct was transformed into the wild-type variety Nipponbare via *Agrobacterium tumefaciens* infection. To generate the plants expressing wild-type and CBM-mutated *BC1*s, *BC1* cDNAs were amplified by PCR. After sequencing confirmation, they were inserted into the binary vector *pCAMBIA1300* between the maize Actin promoter and the Nos terminator. In detail, *BC1OE* construct contains the wild-type *BC1*. *BC1* ^W72A^, *BC1* ^Y46A^, *BC1* ^W66A^, and *BC1* ^Δω^ *GFP* are mutated forms of *BC1*. All the constructs were introduced into the *bc1* mutants. *BC1OE* was also introduced into the wild-type plants.

### Transmission Electron Microscopy

The 2^nd^ internodes from wild type, *bc1*, and transgenic plants were fixed in 2.5% (w/v) glutaraldehyde in 0.1 M PBS (4 mM sodium phosphate, pH 7.2; 200 mM NaCl) at 4°C overnight. The samples were dehydrated through a gradient of ethanol and embedded with Spurr Kit (Sigma). 80-nm ultrathin sections were prepared with an Ultracut E ultramicrotome (Leica) and picked up on formvar-coated copper grids. After post-staining with uranyl acetate and lead citrate, the specimens were observed under a Hitachi H7500 transmission electron microscope. For immunogold labeling, the plant samples were embedded with LR White resin (Sigma). Thin sections (∼100 nm) were cut and mounted on formvar-coated 200-mesh nickel grids. Immunogold labeling was performed as previously described [59]. The grids were stained in 2% aqueous uranyl acetate for 15 min and observed with a Hitachi H7500 transmission electron microscope.

### Generation of BC1 Antibodies

Anti-BC1 polyclonal antibodies were produced in rabbits against a polypeptide containing three repeats of a fragment of the BC1 protein (from residue 242^th^ to 300^th^), and then purified through affinity chromatography. Generations of CESA4, CESA7 and CESA9 specific antibodies were described previously [43]. Anti-ARF1, anti-PIP1s, and anti-XET5 antibodies were purchased from Agrisera. Anti-HSP antibodies were purchased from Protein Innovation (Beijing). The secondary antibody, HRP-conjugated anti-rabbit IgG, was obtained from Sigma.

### Bioinformatics Analysis of BC1

Prediction of the signal peptide and GPI anchor was performed with SignalP [60] (www.cbs.dtu.dk/services/SignalP) and GPI [61] (http://mendel.imp.ac.at/gpi). The Pfam (www.sanger.ac.uk) and SMART (smart.embl-heidelberg.de/smart) searching were used to predict BC1 domain [62], [63]. Multialignment of BC1 and bacterial CBMs were conducted with MultAlin [64] (multalin.toulouse.inra.fr/multalin) and ClustalW2 (www.ebi.ac.uk/Tools/msa/clustalw2). Sequence logo of the BC1's CBM was generated with Weblogo3 (http://weblogo.berkeley.edu/). Coexpression network of *BC1* was analyzed with GenCAT [65] (http://genecat.mpg.de) and the expression profiles of *BC1* and SCW *CESA*s were generated based on the data obtained from RiceGE (http://signal.salk.edu/cgi-bin/RiceGE).

### Protein Blot

The total membrane proteins were extracted from 2 g fresh weight of *bc1*, wild-type, or transgenic plants as previously described [43]. For the Co-IP assay, the young internodes from transgenic rice plants expressing Flag-CESA9 were used to isolate the total membrane proteins. Plant proteins were incubated with anti-FLAG M2 affinity gel (Sigma) overnight at 4°C. The elution before (input) and after (output) immunoprecipitation was separated in the SDS-PAGE gel, transferred onto the nitrocellulose membranes, and analyzed by protein blotting with anti-BC1 and anti-CESA antibodies. To test the solubility of BC1, the membrane fraction was resuspended in 150 µL of either extraction buffer, high salt-buffer (1 M NaCl, 100 mM HEPES-KOH, pH 7.5, 0.3 M sucrose, 5 mM EGTA, and 5 mM EDTA), alkaline buffer (0.1 M Na_2_CO_3_, pH 11, 0.3 M sucrose, 5 mM EGTA, and 5 mM EDTA), or Triton X-100 buffer [1% (v/v) Triton X-100, 100 mM HEPES-KOH, pH 7.5, 0.3 M sucrose, 5 mM EGTA, and 5 mM EDTA] for 1 h. Then, the resulting solution was ultracentrifuged at 100,000 g for 1 h at 4°C to obtain both supernatant and pellet fractions. Each fraction was subjected to immunoblot analysis using the anti-BC1 and anti-CESA9 antibodies at 1∶500 dilutions.

To determine whether BC1 is *N*-glycosylated and GPI-anchored, the total membrane proteins were treated with PNGase F (Biolabs) according to the manufacturer's instructions. Approximately 30 µg of microsomal protein was denatured in glycoprotein denaturing buffer (0.5% SDS, 40 mM DTT) at 100°C for 10 min. After addition of NP-40, G7 reaction buffer (50 mM sodium phosphate, pH 7.5), and two-fold dilutions of PNGase F, the reaction mix was incubated at 37°C for 2 h. Then, the samples were separated using SDS-PAGE and subjected to immunoblot analysis with anti-BC1 (1∶500 dilution) and anti-PIP1s (1∶1000 dilution) antibodies. For testing GPI-anchoring, 30 µg of membrane protein from wild-type plants was resuspended in the extract buffer in the presence or absence of 6 U PLD (Sigma). After 1 h incubation at 37°C, the reactions were ultracentrifuged at 100,000 g for 1 h at 4°C to obtain both supernatant and pellet fractions, and subjected to immunoblot analysis with anti-BC1 and anti-PIP1s antibodies.

Cell wall proteins were prepared as described in [66]. In brief, 5 g of plant tissues was ground in liquid nitrogen, and 20 mL of extraction buffer (5 mM acetate buffer, pH 4.6, 0.4 M sucrose and protease inhibitor cocktail, Invitrogen) was added to the sample. Cell wall pellets were lyophilized after removing the tissue dregs. Cell wall protein fraction was extracted using CaCl_2_ solution (5 mM acetate buffer, pH 4.6, 0.2 M CaCl_2_ and 10 µL protease inhibitor cocktail). Then, the cell wall proteins were extracted by phenol (pH 8.8) and precipitated by adding cold 0.1 M ammonium acetate (made in 100% methanol) overnight. After centrifuging and rinsing with cold methanol and 70% ethanol, the proteins were suspended in 0.1 mL PBS solution and subjected for immunoblot analysis with the antibodies indicated.

### Recombinant Protein Purification

DNA fragments containing the CBM of BC1 (ranging from 23 to 205 amino acid residues) and its mutated forms were inserted in frame into pET-28a. After sequencing conformation, they were transformed into *E. coli* strain BL21 (DE3) pLys competent cells. All proteins were purified from *E. coli* cultures that were harvested after overnight induction with 0.4 mM isopropyl-β-D-thiogalactopyranoside at 16°C. The cells were collected, washed with the binding buffer (20 mM Tris-HCl and 500 mM NaCl, pH 7.4), and disintegrated with ultrasonication. Cell debris was removed by centrifugation. The supernatants were supplied to the Ni-charged column for purification according to the users' manual (Novagen).

### Carbohydrate Microarrays

Carbohydrate microarrays were performed according to the protocol described [24], [67]. The sequentially fractionated rice cell-wall residues, including TFA-insoluble and Updegraff-insoluble residues, were prepared as described [68], [69]. The commercial cell wall products, such as pectin from citrus PGA, MLG from barley, xyloglucan from tamarind, galactomannan from guar, arabinoxylan from wheat, RG from soybean, galactan from lupin, RG-I from potato, and arabinan from sugar beet were purchased from Megazyme (http://www.megazyme.com), and xylan from birchwood, microgranular cellulose, and 20 µm microcrystalline cellulose were from Sigma (http://www.Sigma.com). Avicel pH 101 cellulose powder was purchased from Fluka (http://www.fluka.org). The above substrates were dissolved in PBS buffer at 1 mg/mL and 0.1 mg/mL to prepare the stocks. Five microliters of each stock was spotted on nitrocellulose membrane (PALL). After drying, these membranes were incubated with 5% non-fat milk for 2 h at room temperature and probed with the CBM of BC1 (50 µg/mL), JIM5 (1∶200), LM11 (1∶200), CBM3a (1∶100), and CBM28 (1∶100) proteins or antibodies. Then, the membrane was probed with anti-His antibody (1∶1000, Sigma) and the secondary antibodies goat anti-mouse IgG or goat anti-rat IgG to horseradish peroxidase. The signals were detected on X-ray film and quantified with GelQuantNet (biochemlabsolutions.com). The values were calculated by the ratios of signal intensity of the spots to that of the weakest visible spots on the same blot.

### ELISA

ELISA assays were performed according to a previous report [24]. Protein solutions were incubated with 100 µg of updegraff insoluble residues from plants and commercial cellulose products for 1 h at 4°C. The supernatants after the binding assay were applied to 96-well EIA/RIA flat-well high binding plates (Costar) (100 µL/per well) and incubated overnight. Sodium carbonate solution was used as a blank. The samples and the blank were blocked with 300 µL of 5% non-fat milk in PBST (PBS with 0.1% Tween 20) for at least 6 h. The wells were washed extensively with PBST and incubated with rabbit anti-His antibody (Sigma) in 5% milk overnight. After extensive washing, the wells were incubated with the secondary antibody, rabbit anti-mouse to horseradish peroxidase (Thermo Scientific) in 5% milk. The wells were finally incubated with 150 µL of tetramethylbenzidine (Sigma), and the absorbance at 450 nm was recorded using a plate reader (Immunosorbent Detector; GENios) after applying 35 µL of 2 N sulfuric acid to stop the reaction. The binding activity was shown as the percentage of bound protein to total proteins added.

### Binding Activity Assays of the CBM in BC1

Different amounts of recombinant CBM were added and incubated with 100 µg of updegraff insoluble residues for 1 h at 4°C. After centrifugation, the supernatant was subjected to ELISA assay. A dissociation constant value for CBM to rice crystalline cellulose was calculated by fitting a hyperbolic function to the ELISA data as free protein and the total mount added minus the free protein as bound protein, and by using Origin v8.0 software (Origin). To examine the binding affinity of the point-mutated CBMs, same amount of the recombinant proteins were incubated with 100 µg of cellulose powder (20 µm, Sigma), and ELISA assay was performed as described above. The relative binding activity was determined by the ratio of bound proportion of the mutated CBM to that of wild-type CBM (which was considered as 1).

### Cell Wall Structure Analysis by XRD

The 3^rd^ internodes from 4-month-old and development-matched rice plants were collected and ground in liquid nitrogen for preparation of the cell wall residues. XRD analysis was performed accordingly [20]. In detail, Synchrotron X-ray diffraction experiments were performed on a six-circle diffractometer (Huber 5020) in the Beijing Synchrotron Radiation Facility Center using CuKα radiation with a wave length of λ = 0.154 nm. An X-ray beam with a photon energy of 8.051 keV was focused on the plant samples. X-ray diffractograms were collected by a scintillator detector Huber9910 that was set at a distance of 45 mm from the sample. Data were recorded and integrated using the Origin v8.0 software (Origin) to generate the 2D diffraction image. The relative crystallinity index (RCI) was determined as follows: RCI = (*I_002_−I_am_*)/*I_002_*×100, where *I_002_* represents both crystalline and amorphous materials (2*θ* = 22.7°) and *I_am_* represents amorphous materials (2*θ* = 18°) by using commercial crystalline cellulose as a control. Scherrer equation analysis was performed to obtain the crystallite size of rice samples by fitting a Gauss peak to the 200 reflection.

### Cell Wall Analysis

The monosaccharide composition was determined by GC-MS (Agilent), as described previously [43]. In brief, 2 mg of destarched AIRs were hydrolyzed in 2 M trifluoroacetic acid (TFA) at 121°C for 90 min. The supernatants were air dried and reduced with sodium borohydride (10 mg/mL in 1 M ammonium hydroxide). The generated alditol acetates were extracted in ethyl acetate and analyzed by an Agilent 7890 GC system equipped with a 5975C MSD (Agilent).

For crystalline cellulose analysis, the remains after TFA treatment were hydrolyzed in Updegraff reagent. The cooled pellets were washed and hydrolyzed with 72% sulfuric acid. The cellulose content was quantified by the anthrone assay [68].

### Immunostaining and Fluorescent Cellulose Dye Treatments

The Updegraff-insoluble rice residues were spotted onto poly-lysine-treated glass slides. After drying, the slide was blocked in PBS buffer containing 1% BSA and incubated with PBS buffer containing 20 µg purified CBM of BC1 or IIP4, a known nuclear protein (a negative control) [41]. Then, the slide was subjected to immunostaining as described [69]. For immunolabeling the S4B- and Calcofluor-stained cellulose residues, the Updegraff-insoluble residues were pre-stained with 0.01% S4B solution (Sigma) or 0.005% calcofluor solution (Sigma). Then, the stained and unstained residues were spotted onto the poly-lysine mounted slides for immunochemical staining. The primary antibody, anti-His antibody (1∶1000), and the secondary antibodies, fluorescein-5-isothiocyanate (FITC)-conjugated rabbit anti-mouse IgG (1∶500) or Cy3-conjugated rabbit anti-mouse IgG (1∶500), were purchased from Sigma. To test the in vivo effect of the cellulose dyes on plant growth, the germinated seeds of wild type and *bc1* plants were transferred onto MS media containing various concentrations of S4B and Calcofluor dyes and cultivated for 60 h. The length of the primary root was recorded every 12 h.

### Gene Expression

For real-time PCR analysis, the 3^rd^ internodes were collected for RNA extraction with Plant RNA Reagent (Invitrogen). Total RNAs were used to synthesize cDNA with a Reverse Transcription System (Promega). qRT-PCR was performed on a cycler apparatus (Bio-Rad) with FastStart Universal SYBR Green Master (Roche) using the following program: 94°C for 4 min, 40 cycles of 94°C for 30 s, 58°C for 30 s, and 72°C for 30 s. *eEF1α* and *UBQ5* were used as internal controls for normalization. Data were presented as the mean values of three biological repeats. The primers for gene expression assay are shown in Table S2.

## Supporting Information

Figure S1 Bioinformatics analysis of BC1. (A) Schematic structure of BC1, showing the signal peptide (SP) at the N-terminus and the ω site that will attach GPI anchor at the C-terminus. The mutation site in *bc1* that causes premature terminated BC1 is indicated by a blue box, and the antigen for BC1 antibody production is shown by an orange box. (B) Alignment of the amino acid sequence (242^th^ to 300^th^) used for generation of BC1 antibody with that of the BC1-like members in rice. The conserved amino acids are shown in shadow. (C) Hydropathy plot and the ω site prediction of BC1.
(TIF)

Figure S2 Alignment of the CBM in BC1 with its homologs in plants and bacteria. (A) The partial CBM sequences from rice and Arabidopsis. Letters at left indicate the protein name. The conserved amino acids are shown in shadow. The triangles indicate the conserved aromatic residues studied here. (B) Alignment of the CBM from BC1 and bacteria. The conserved residues are shown in shadow, in which the conserved aromatic residues studied by McLean et al. [42] are shown in blue; and the conserved aromatic residues studied here are shown in red. *CfiCenA*, *Cellulomonas fimi* endoglucanase A; *CfiCex*, *Cellulomonas fimi* xylanase A; *PflXynA*, *Pseudomonas fluorescens* xylanase A; *CloCelA*, *Clostridium thermocellum* endoglucanase A; *CfiXylD*, *Cellulomonas fimi* xylanase D; *SliAxeA*, *Streptomyces lividans* acetylxylan esterase A; *TfuXynA*, *Thermomonospora fusca* xylanase A.
(TIF)

Figure S3 Carbohydrate microarray. (A) Spotting grid for various carbohydrates that derived from different sources. Each grid contains two concentrations of the spotting solution (1 mg/mL and 0.1 mg/mL). (B) Microarrays via incubation with BC1-CBM, JIM5 (anti-pectin), MLG (anti β-1,3-1,4-glucan), CBM3a (anti-crystalline cellulose), LM11 (anti-xylan), and CBM28 (anti-amorphous cellulose) primary antibodies, and the horseradish peroxidase-coupled secondary antibodies. (C) Blotting the rice TFA-insoluble residues with the CBM and sugar antibodies as indicated.
(TIF)

Figure S4 The aromatic amino acids are essential for cellulose binding. (A) Sequence logo assessment of residues in the CBM of BC1 and COBLs in rice, Arabidopsis, poplar, and maize illustrates the location and conservation of the aromatic amino acids. Amino acids are colored according to the chemical properties: hydrophobic and aromatic residues are in blue, hydrophilic ones are shown in black, and neutral ones are in green. Red boxes indicate the amino acids selected for mutagenesis analysis in this study. (B) Changing the three residues highlighted in blue to the ones highlighted in red. (C) Protein blotting the purified recombinant proteins with anti-His antibody, to monitor the amount of purified proteins added for binding activity assay.
(TIF)

Figure S5 Examination of secondary cell wall structure in rice internodes. (A and B) TEM micrographs of wild-type sclerenchyma cell walls. (C and D) TEM micrographs of *bc1* sclerenchyma cell walls. The electron-dense stained materials are indicated by red arrows. S1 to S3, three layers of secondary cell wall; SCW, secondary cell wall; PCW, primary cell wall. Bars = 5 µm in (A and C) and 1 µm in (B and D).
(TIF)

Figure S6 Examination of the affinity of recombinant CBMs for rice internodes. (A–F) Immunogold labeling of rice internode-cross sections with CBM3a (A), CBM28 (B), BC1-CBM (C), and three CBM-mutated variants (D–F), respectively. Bars = 2 µm.
(TIF)

Figure S7 GPI-Substitution is essential for BC1 delivery. (A) Protein blotting of BC1-GFP with the indicated antibodies in the protein extracts from plants expressing the *BC1* ^Δω^ *GFP* with and without PNGase F and PLD treatments. TM, total membrane. ARF1 served as a loading control. (B) Mannitol-induced plasmolysis to examine BC1^Δω^GFP in the root cells of transgenic plants. (C) Cellulose content of the 2^nd^ internodes of wild type, *bc1*, and the *BC1* ^Δω^ *GFP* transgenic plants. Data of mean ± SE (*n* = 3, **P<0.01 by Student's *t*-test). Bar = 10 µm in (B).
(TIF)

Figure S8 *BC1* is highly co-expressed with SCW *CESA*s. (A) Co-expression network of *BC1*. *BC1* is shown in blue, and the tightly co-expressed genes are shown in green. (B) Expression profiles of *BC1* and three SCW *CESA*s generated from the published microarray data.
(TIF)

Figure S9 BC1 affects cellulose crystallinity. (A and B) The representative two-dimensional scattering images of the 3^rd^ internodes from the indicated 4-month old rice plants generated by wide-angle XRD. RCI was obtained using Bragg-Brentono reflective geometries. (C and D) Primary root length of wild-type and *bc1* plants grown in the media containing the indicated concentrations of S4B (C) and Calcofluor (D) for 60 h.
(TIF)

Figure S10 *bc1* has altered cellulose crystallinity status. (A–D) Immuno-staining of the unstained or pre-stained rice crystalline cellulose (0.005% Calcofluor, w/v) with the CBM by using anti-His and anti-Cy3 as the primary and secondary antibodies. Bar = 100 µm. (E) Effects of Calcofluor on root growth in wild-type and *bc1* seedlings. Primary root length was measured at the indicated time. Data of mean ± SE (*n*≥15). Square brackets indicate the varied responses to one concentration of Calcofluor between wild-type and *bc1* seedlings.
(TIF)

Table S1 The noncellulosic sugar composition of the 3^rd^ internodes from wild type, *bc1*, and relevant transgenic plants (µg mg^−1^ AIR).
(DOC)

Table S2 List of the primers used in this study.
(DOC)
