# Supplementary figures and images for: Brittle Culm1, a COBRA-Like Protein, Functions in Cellulose Assembly through Binding Cellulose Microfibrils
Source: PLoS Genet. 2013 Aug 22;9(8):e1003704. doi: 10.1371/journal.pgen.1003704 (PMC3749933; doi:10.1371/journal.pgen.1003704)

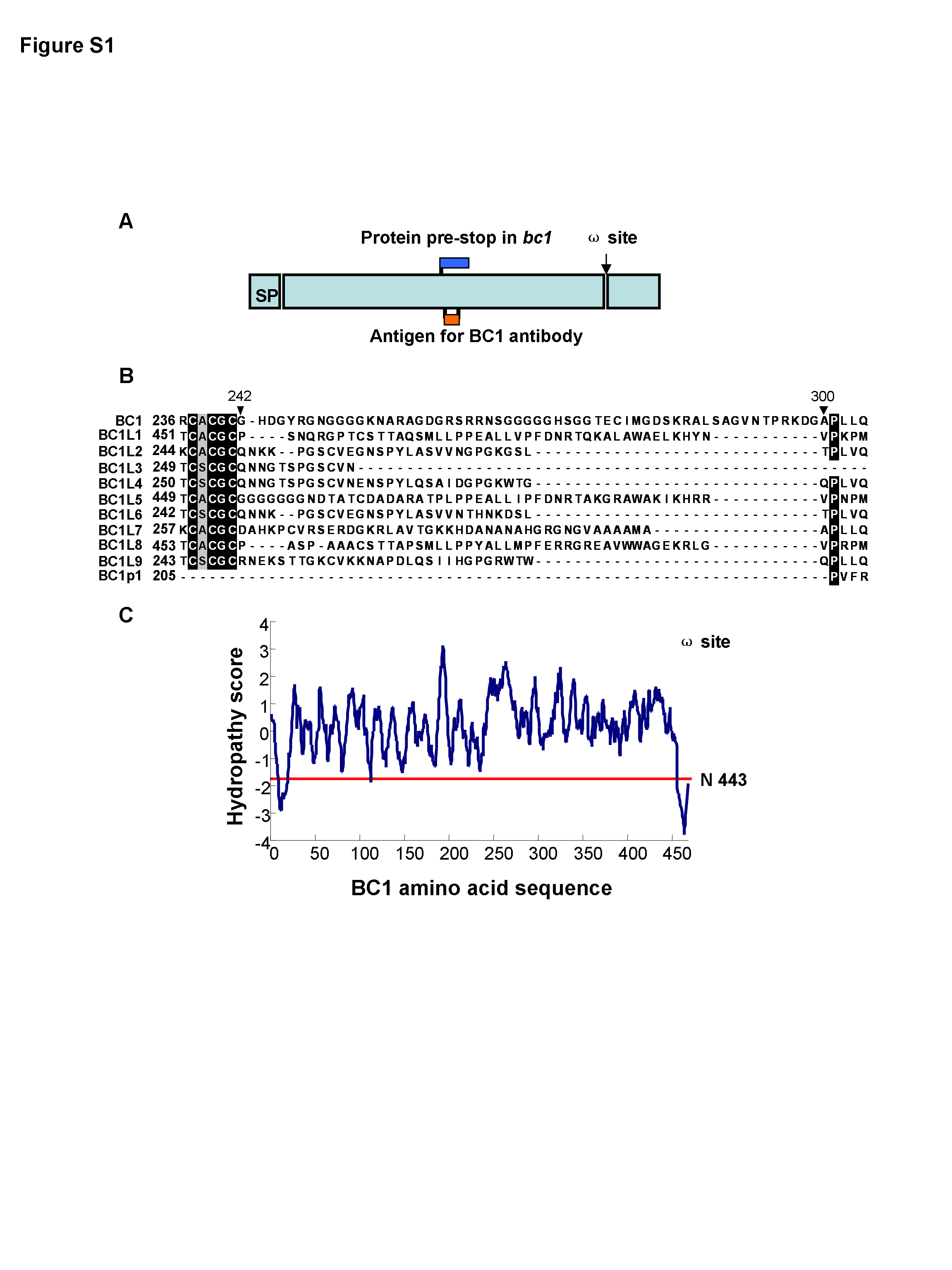

Supplement: Figure S1 — Bioinformatics analysis of BC1. (A) Schematic structure of BC1, showing the signal peptide (SP) at the N-terminus and the ω site that will attach GPI anchor at the C-terminus. The mutation site in bc1 that causes premature terminated BC1 is indicated by a blue box, and the antigen for BC1 antibody production is shown by an orange box. (B) Alignment of the amino acid sequence (242th to 300th) used for generation of BC1 antibody with that of the BC1-like members in rice. The conserved amino acids are shown in shadow. (C) Hydropathy plot and the ω site prediction of BC1. (TIF) [file pgen.1003704.s001.tif]

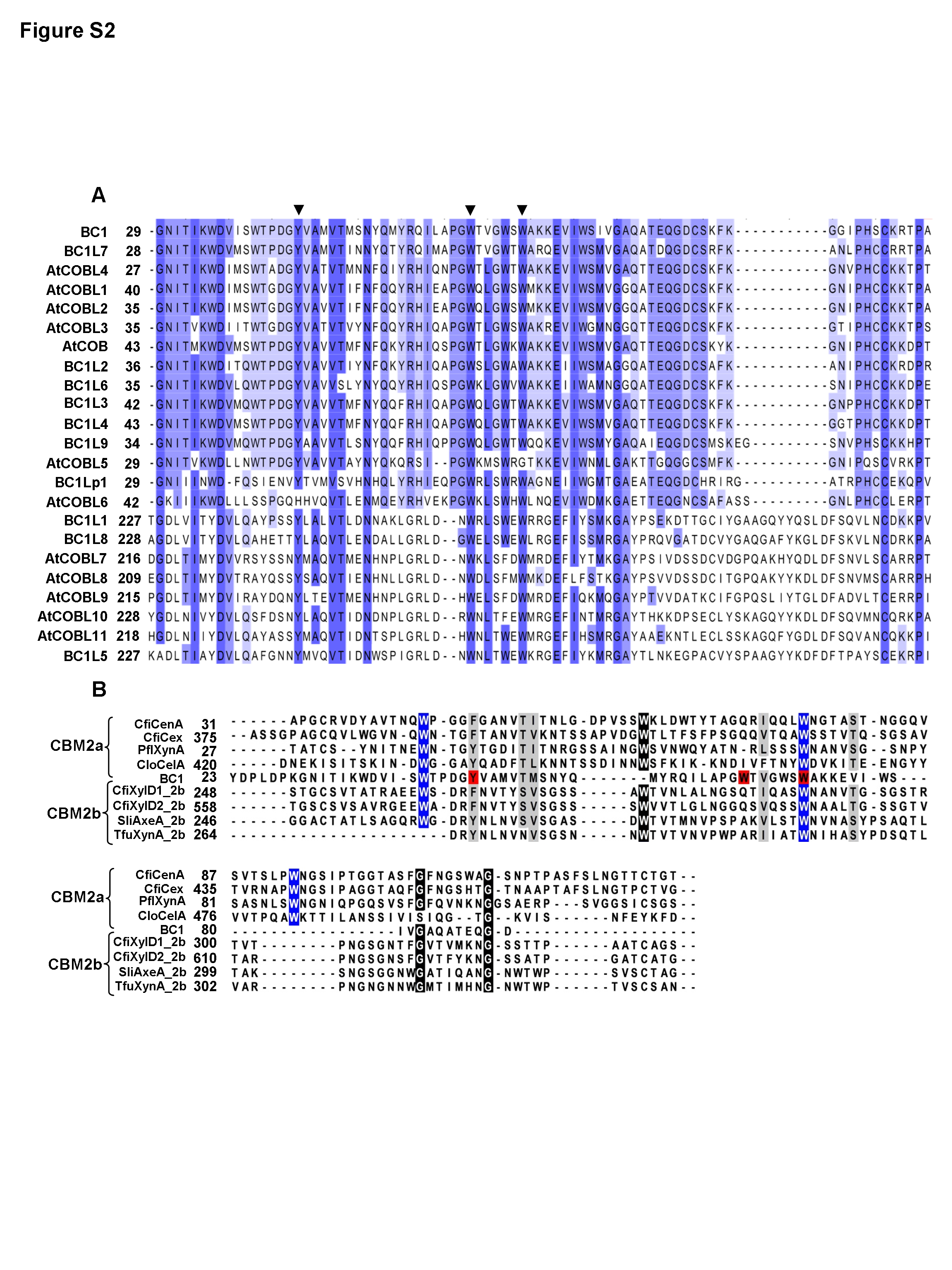

Supplement: Figure S2 — Alignment of the CBM in BC1 with its homologs in plants and bacteria. (A) The partial CBM sequences from rice and Arabidopsis. Letters at left indicate the protein name. The conserved amino acids are shown in shadow. The triangles indicate the conserved aromatic residues studied here. (B) Alignment of the CBM from BC1 and bacteria. The conserved residues are shown in shadow, in which the conserved aromatic residues studied by McLean et al. [42] are shown in blue; and the conserved aromatic residues studied here are shown in red. CfiCenA, Cellulomonas fimi endoglucanase A; CfiCex, Cellulomonas fimi xylanase A; PflXynA, Pseudomonas fluorescens xylanase A; CloCelA, Clostridium thermocellum endoglucanase A; CfiXylD, Cellulomonas fimi xylanase D; SliAxeA, Streptomyces lividans acetylxylan esterase A; TfuXynA, Thermomonospora fusca xylanase A. (TIF) [file pgen.1003704.s002.tif]

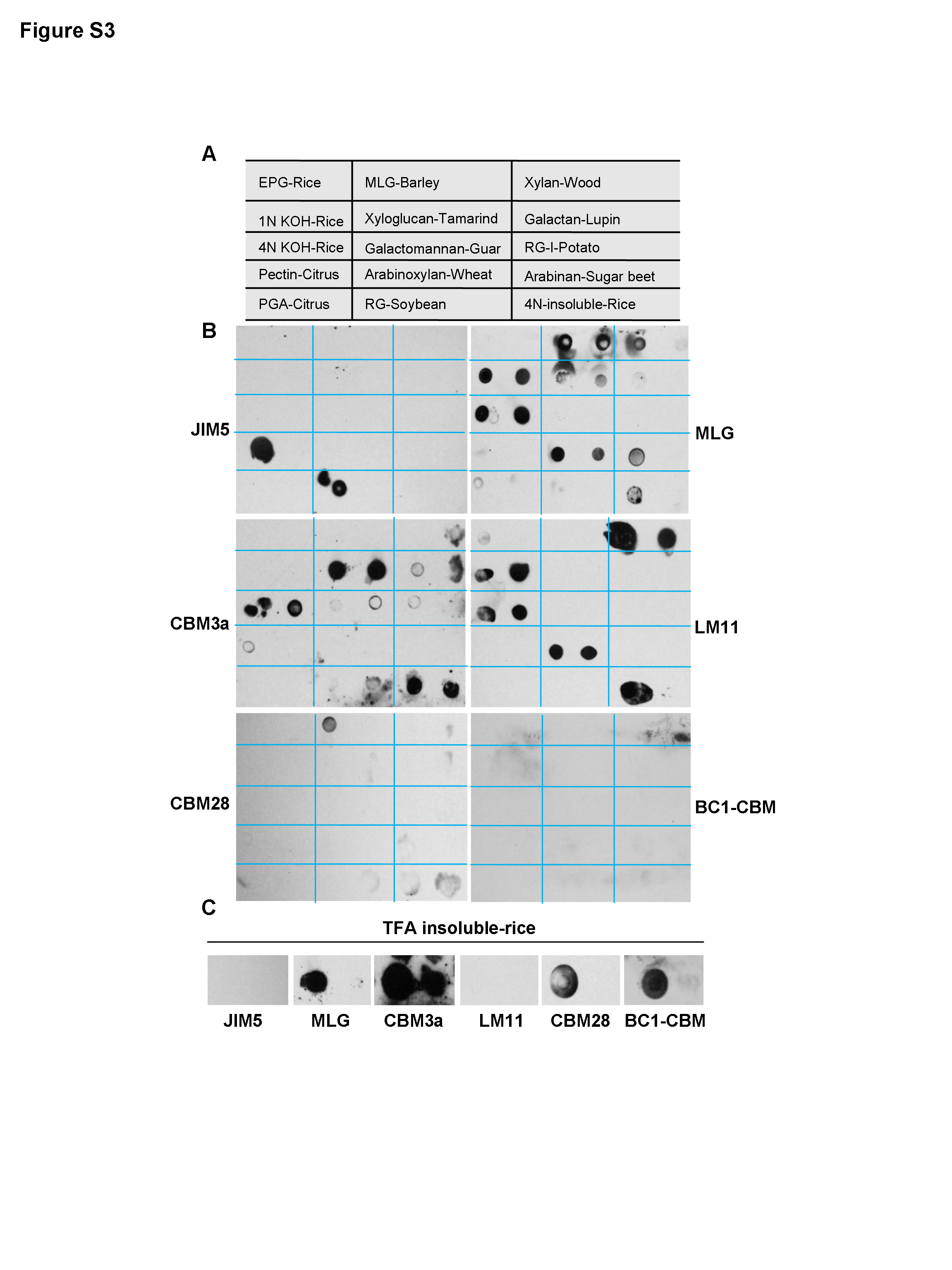

Supplement: Figure S3 — Carbohydrate microarray. (A) Spotting grid for various carbohydrates that derived from different sources. Each grid contains two concentrations of the spotting solution (1 mg/mL and 0.1 mg/mL). (B) Microarrays via incubation with BC1-CBM, JIM5 (anti-pectin), MLG (anti β-1,3-1,4-glucan), CBM3a (anti-crystalline cellulose), LM11 (anti-xylan), and CBM28 (anti-amorphous cellulose) primary antibodies, and the horseradish peroxidase-coupled secondary antibodies. (C) Blotting the rice TFA-insoluble residues with the CBM and sugar antibodies as indicated. (TIF) [file pgen.1003704.s003.tif]

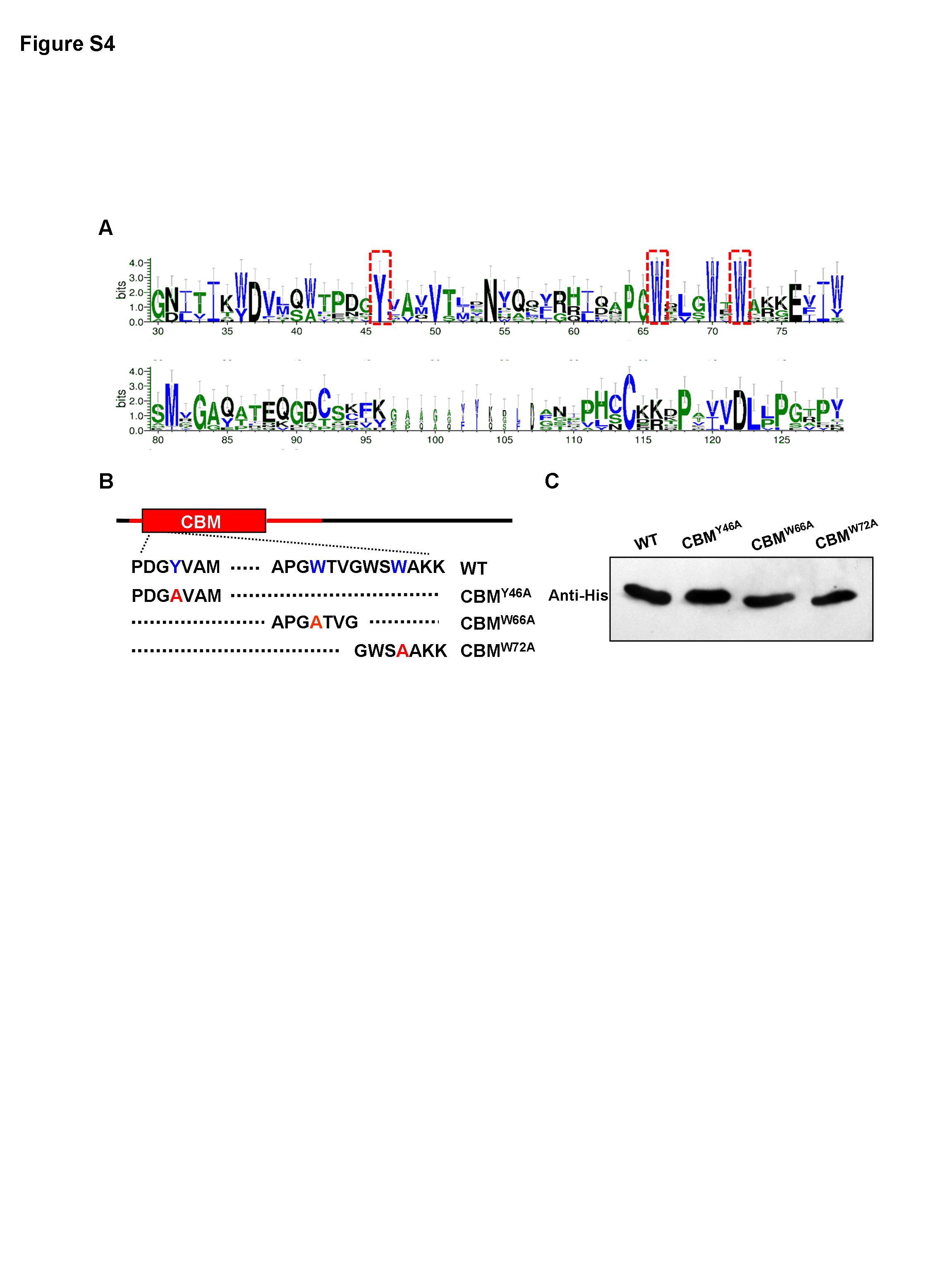

Supplement: Figure S4 — The aromatic amino acids are essential for cellulose binding. (A) Sequence logo assessment of residues in the CBM of BC1 and COBLs in rice, Arabidopsis, poplar, and maize illustrates the location and conservation of the aromatic amino acids. Amino acids are colored according to the chemical properties: hydrophobic and aromatic residues are in blue, hydrophilic ones are shown in black, and neutral ones are in green. Red boxes indicate the amino acids selected for mutagenesis analysis in this study. (B) Changing the three residues highlighted in blue to the ones highlighted in red. (C) Protein blotting the purified recombinant proteins with anti-His antibody, to monitor the amount of purified proteins added for binding activity assay. (TIF) [file pgen.1003704.s004.tif]

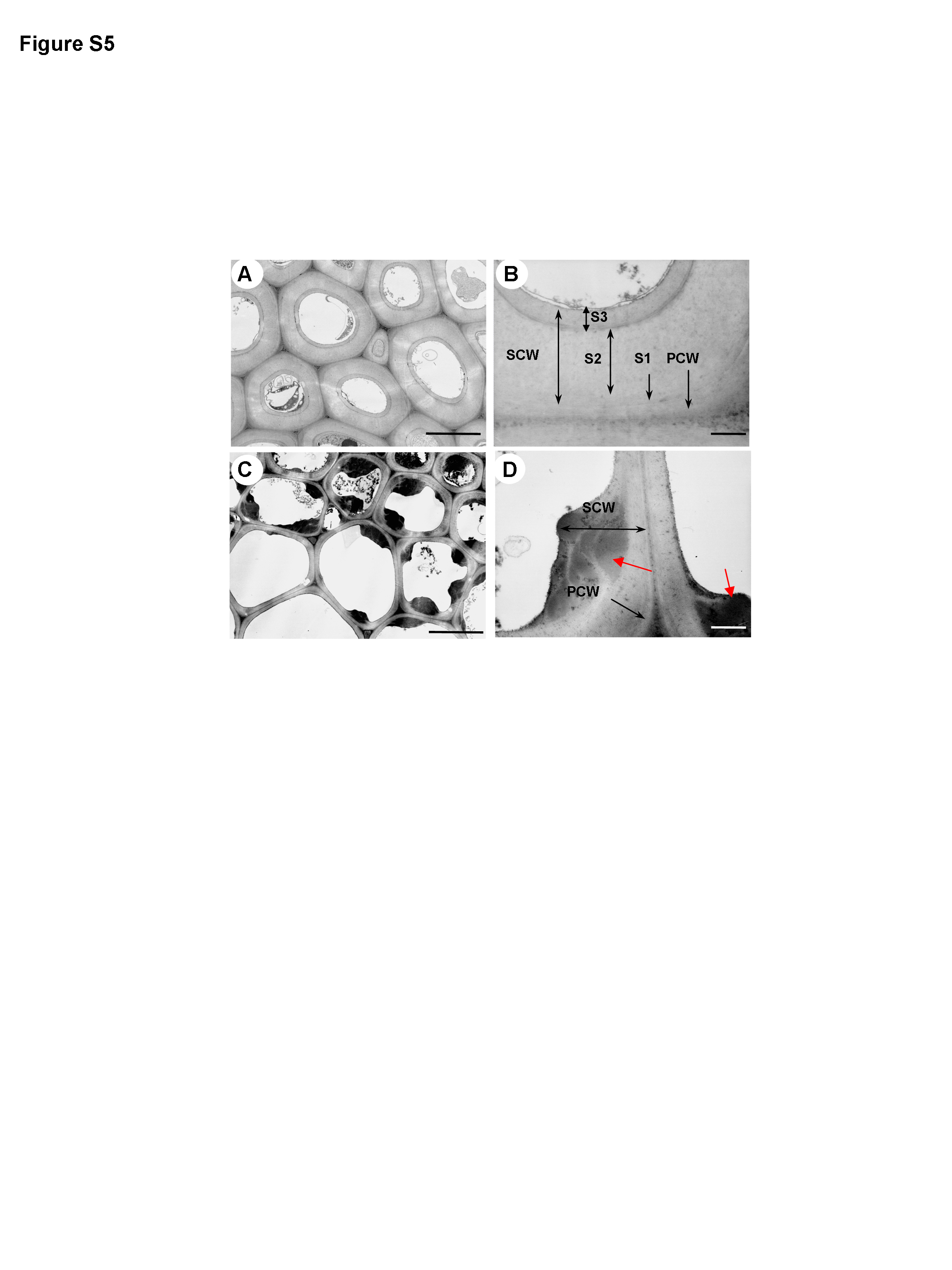

Supplement: Figure S5 — Examination of secondary cell wall structure in rice internodes. (A and B) TEM micrographs of wild-type sclerenchyma cell walls. (C and D) TEM micrographs of bc1 sclerenchyma cell walls. The electron-dense stained materials are indicated by red arrows. S1 to S3, three layers of secondary cell wall; SCW, secondary cell wall; PCW, primary cell wall. Bars = 5 µm in (A and C) and 1 µm in (B and D). (TIF) [file pgen.1003704.s005.tif]

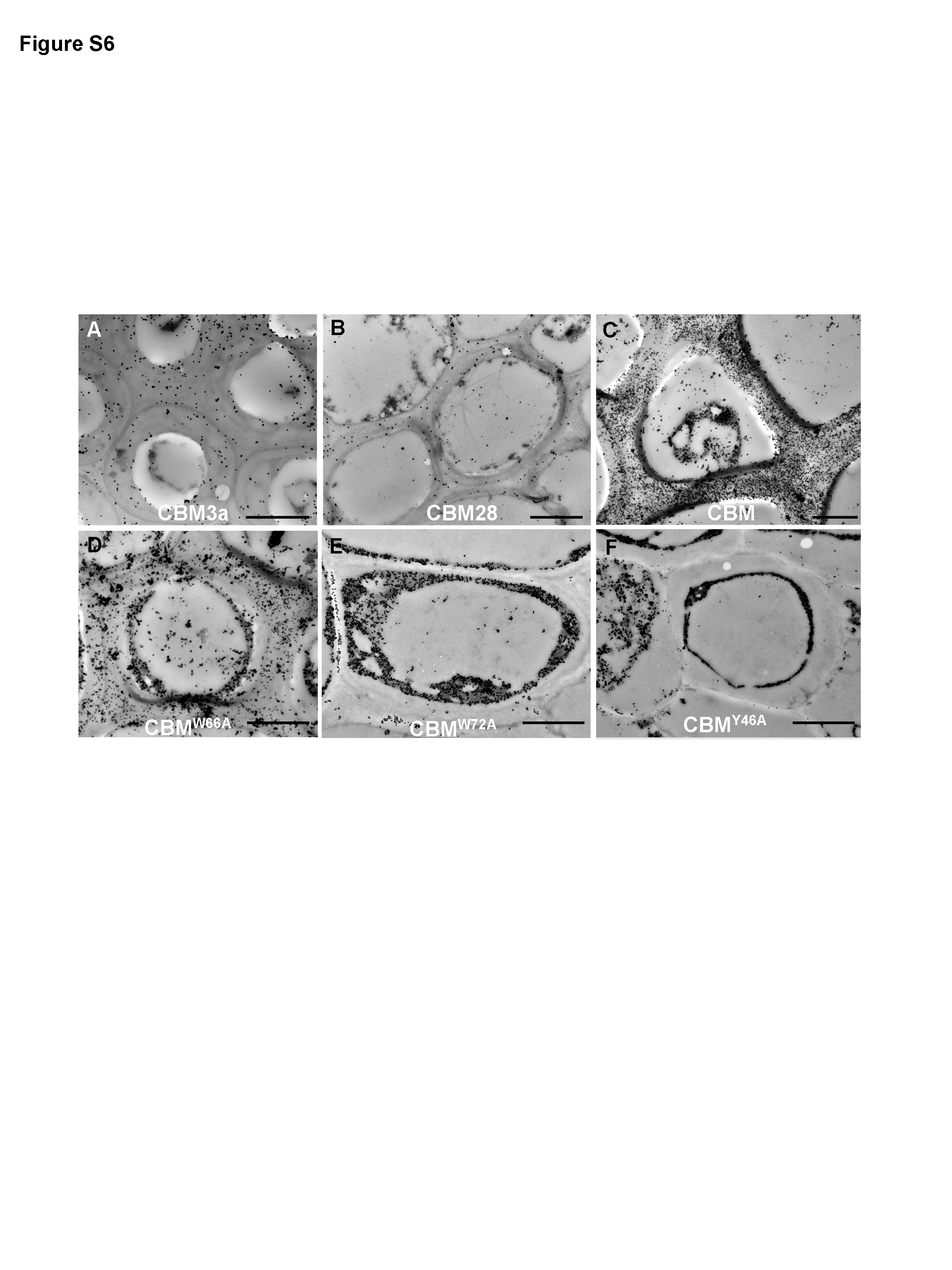

Supplement: Figure S6 — Examination of the affinity of recombinant CBMs for rice internodes. (A–F) Immunogold labeling of rice internode-cross sections with CBM3a (A), CBM28 (B), BC1-CBM (C), and three CBM-mutated variants (D–F), respectively. Bars = 2 µm. (TIF) [file pgen.1003704.s006.tif]

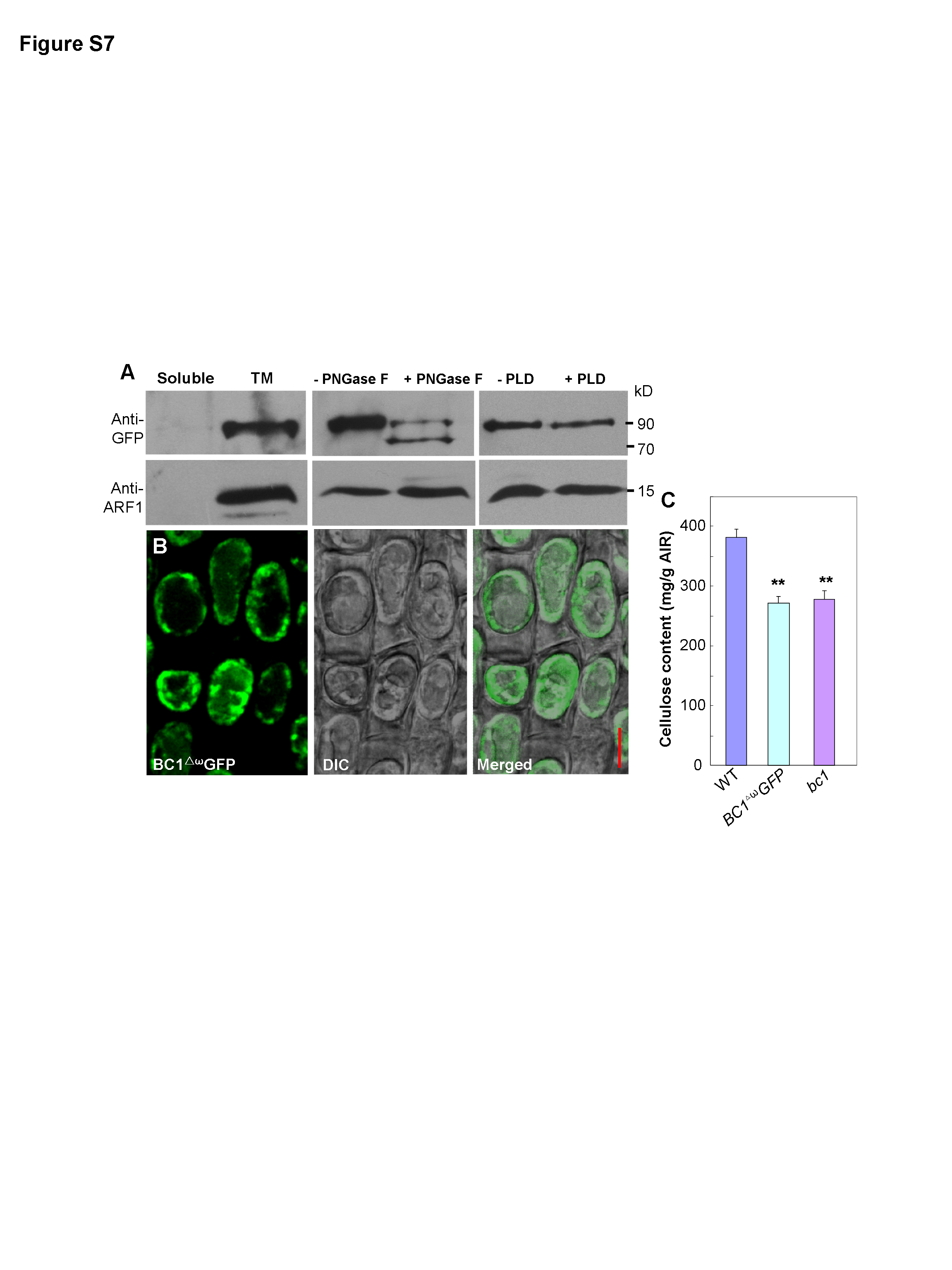

Supplement: Figure S7 — GPI-Substitution is essential for BC1 delivery. (A) Protein blotting of BC1-GFP with the indicated antibodies in the protein extracts from plants expressing the BC1 Δω GFP with and without PNGase F and PLD treatments. TM, total membrane. ARF1 served as a loading control. (B) Mannitol-induced plasmolysis to examine BC1ΔωGFP in the root cells of transgenic plants. (C) Cellulose content of the 2nd internodes of wild type, bc1, and the BC1 Δω GFP transgenic plants. Data of mean ± SE (n = 3, **P<0.01 by Student's t-test). Bar = 10 µm in (B). (TIF) [file pgen.1003704.s007.tif]

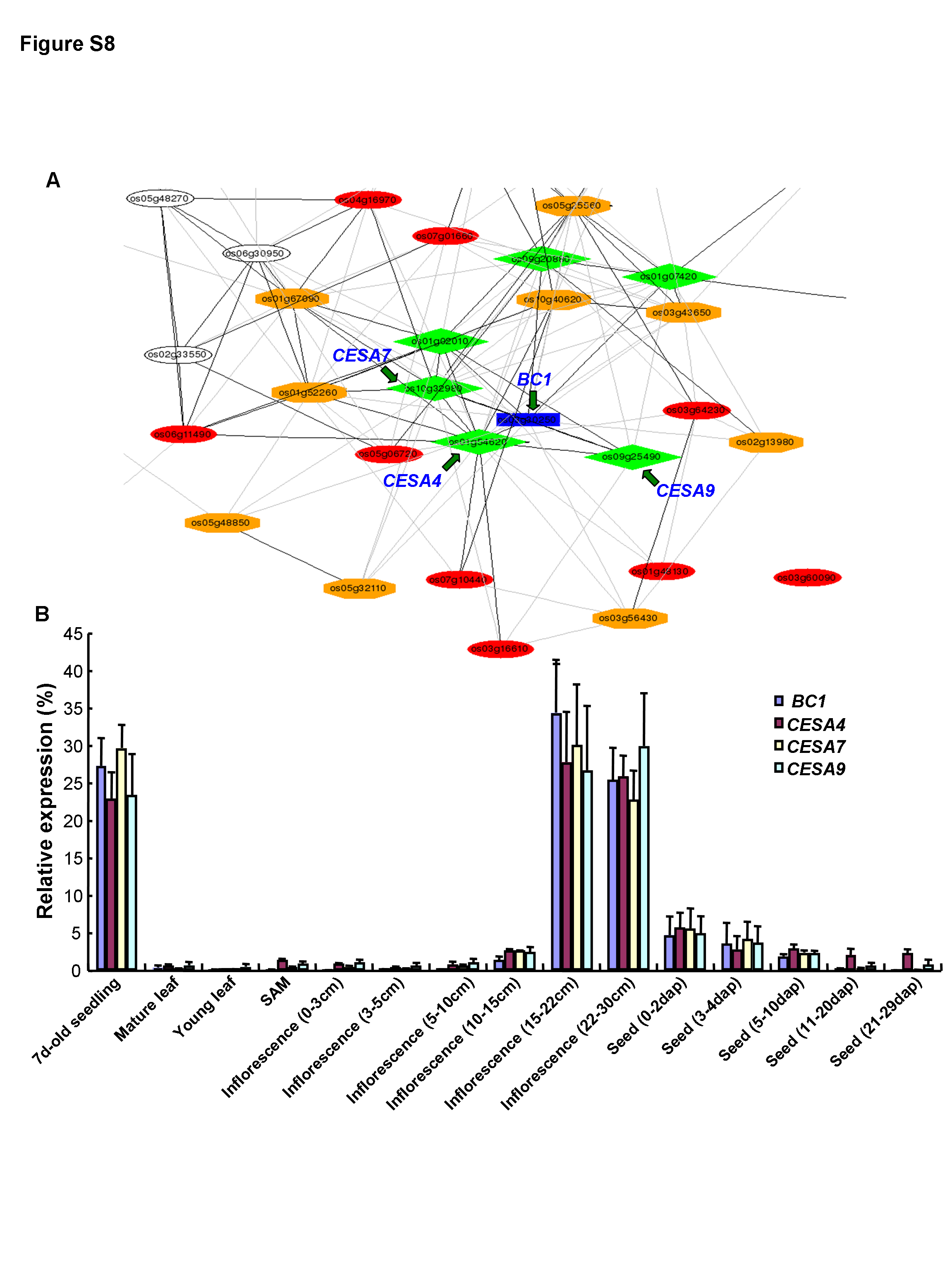

Supplement: Figure S8 — BC1 is highly co-expressed with SCW CESAs. (A) Co-expression network of BC1. BC1 is shown in blue, and the tightly co-expressed genes are shown in green. (B) Expression profiles of BC1 and three SCW CESAs generated from the published microarray data. (TIF) [file pgen.1003704.s008.tif]

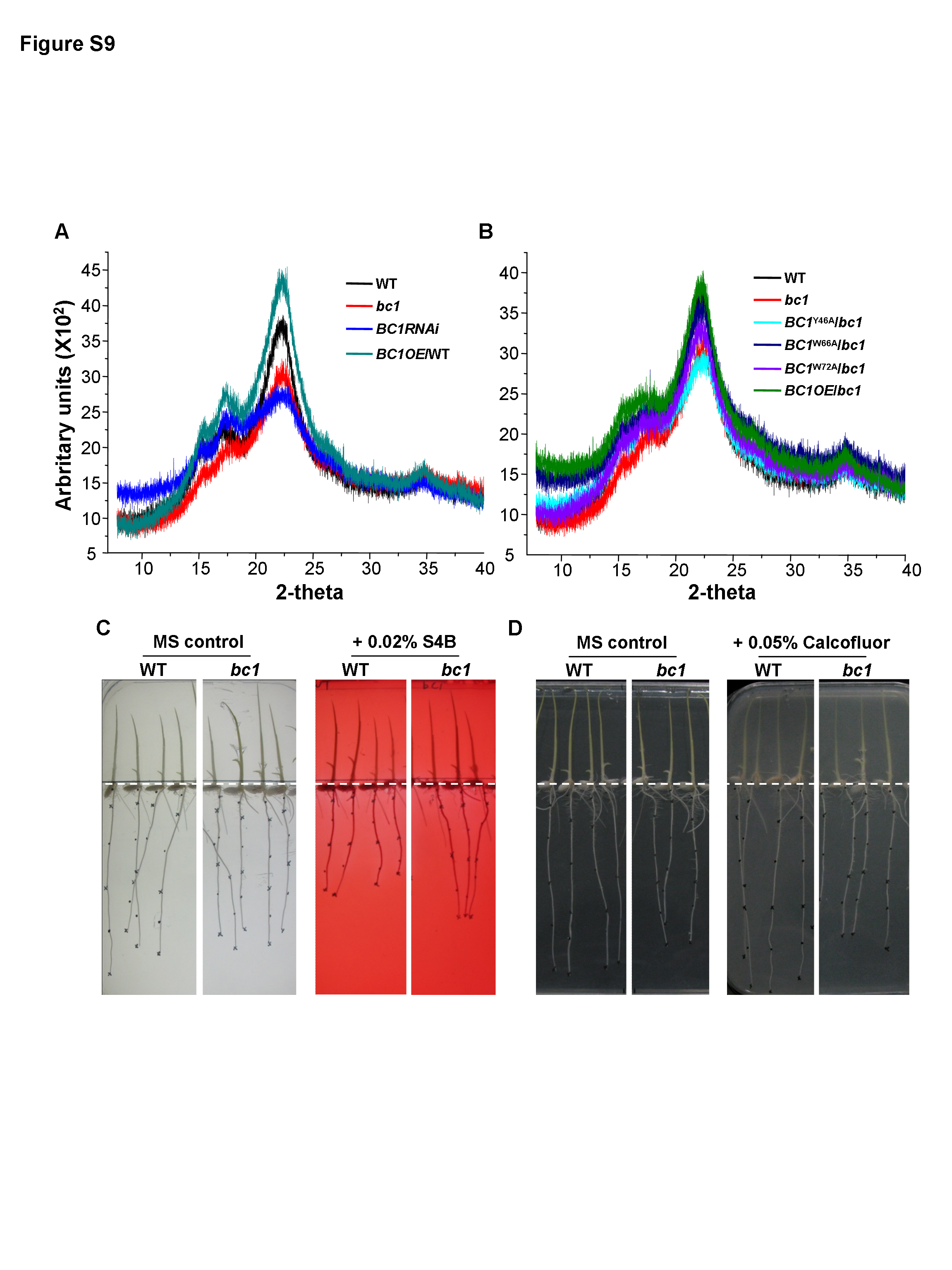

Supplement: Figure S9 — BC1 affects cellulose crystallinity. (A and B) The representative two-dimensional scattering images of the 3rd internodes from the indicated 4-month old rice plants generated by wide-angle XRD. RCI was obtained using Bragg-Brentono reflective geometries. (C and D) Primary root length of wild-type and bc1 plants grown in the media containing the indicated concentrations of S4B (C) and Calcofluor (D) for 60 h. (TIF) [file pgen.1003704.s009.tif]

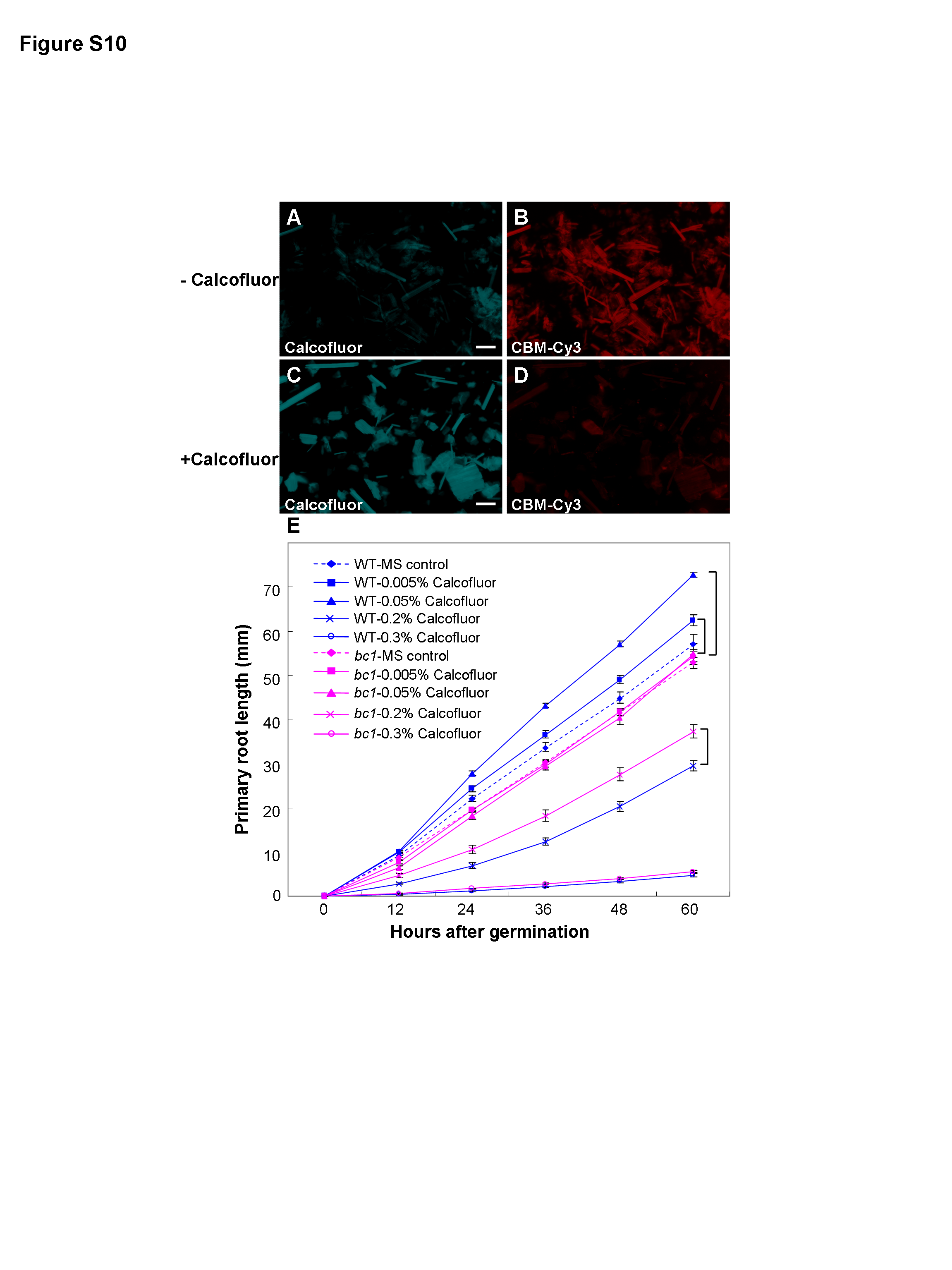

Supplement: Figure S10 — bc1 has altered cellulose crystallinity status. (A–D) Immuno-staining of the unstained or pre-stained rice crystalline cellulose (0.005% Calcofluor, w/v) with the CBM by using anti-His and anti-Cy3 as the primary and secondary antibodies. Bar = 100 µm. (E) Effects of Calcofluor on root growth in wild-type and bc1 seedlings. Primary root length was measured at the indicated time. Data of mean ± SE (n≥15). Square brackets indicate the varied responses to one concentration of Calcofluor between wild-type and bc1 seedlings. (TIF) [file pgen.1003704.s010.tif]
