# Supplementary material for: Brittle Culm1, a COBRA-Like Protein, Functions in Cellulose Assembly through Binding Cellulose Microfibrils
Source: PLoS Genet. 2013 Aug 22;9(8):e1003704. doi: 10.1371/journal.pgen.1003704 (PMC3749933; doi:10.1371/journal.pgen.1003704)
Supplement: Table S1 — The noncellulosic sugar composition of the 3rd internodes from wild type, bc1, and relevant transgenic plants (µg mg−1 AIR). (DOC) [file pgen.1003704.s011.doc]

**Table S1.** The Noncellulosic Sugar Composition of the 3^rd^ Internodes from Wild Type, *bc1*, and Relevant Transgenic Plants (μg mg^-1^ AIR)

| Residues | Rhamnose | Fucose | Arabinose | Xylose | Mannose | Galactose | Glucose |
| --- | --- | --- | --- | --- | --- | --- | --- |
| WT | 0.6 ± 0.3 | 0.2 ± 0.2 | 28.7 ± 0.2 | 199.3 ± 3.3 | 2.3 ± 0.1 | 19.1 ± 0.3 | 80.6 ± 1.3 |
| *bc1* | 0.3 ± 0.3 | 0.2 ± 0.2 | 25.2 ± 1.1 | 255.6 ± 7.3* | 2.2 ± 0.1 | 17.5 ± 0.6 | 82.8 ± 3.6 |
| *BC1RNAi*^1^ | ND | 0.4 ± 0.3 | 32.7 ± 0.8* | 361.3 ± 5.0* | 2.2 ± 0.1 | 15.4 ± 0.9 | 35.9 ± 2.7* |
| *BC1OE*/WT^1^ | 0.5 ± 0.3 | 0.2 ± 0.2 | 25.5 ± 0.6 | 185.9 ± 5.4* | 2.2 ± 0.1 | 17.0 ± 0.3 | 65.7 ± 1.2 |
| *BC1OE*/*bc1*^2^ | 0.5 ± 0.3 | 0.2 ± 0.2 | 18.2 ± 0.9* | 214.3 ± 3.0 | 2.0 ± 0.1 | 7.1 ± 0.6* | 39.0 ± 1.5* |
| *BC1*^Y46A^/*bc1*^2^ | ND | 0.6 ± 0.2 | 26.3 ± 0.4 | 245.8 ± 7.9* | 2.1 ± 0.1 | 17.3 ± 0.5 | 75.9 ± 3.6 |
| *BC1*^W66A^/*bc1*^2^ | 0.3 ± 0.3 | ND | 21.4 ± 0.7* | 202.3 ± 3.7 | 2.2 ± 0.1 | 12.4 ± 0.4* | 64.5 ± 1.5 |
| *BC1*^W72A^/*bc1*^2^ | 0.3 ± 0.3 | 0.4 ± 0.2 | 22.7 ± 0.3* | 258.1 ± 4.3* | 2.0 ± 0.0 | 11.7 ± 0.2* | 55.1 ± 2.1* |
| As determined by GC-MS analysis of alditol acetate derivatives of alcohol-insoluble residues (AIRs). *Significantly different (*t* test at *P* < 0.01) with respect to wild type (*n* = 3) ± SE.  ^1^, ^2^ Expressing the indicated constructs in wild-type and *bc1* mutant plants, respectively.  ND, not detected. | | | | | | | |
