# Supplementary material for: Brittle Culm1, a COBRA-Like Protein, Functions in Cellulose Assembly through Binding Cellulose Microfibrils
Source: PLoS Genet. 2013 Aug 22;9(8):e1003704. doi: 10.1371/journal.pgen.1003704 (PMC3749933; doi:10.1371/journal.pgen.1003704)
Supplement: Table S2 — List of the primers used in this study. (DOC) [file pgen.1003704.s012.doc]

**Table S2.** List of the Primers Used in This Study

| Markers | Forward primer | Reverse primer |
| --- | --- | --- |
| *BC1*-qRT | CTTTGAAATTGCCTGATAGA | AAAGTTTGTGGTGTGATTT |
| *CESA4*-qRT | CTCCGAGACCACCACCACCAAC | ACCCATCGTCTTCGTCGCATTAG |
| *CESA7*-qRT | AAGCCATGCGGGGTCTCGTG | CATCCATCCGGTCATCCCTCTTG |
| *CESA9*-qRT | ATCGCGCTCTTCATCTCCATCTTC | ACTGCTCGTTCCTCCACCACTCC |
| *BC1RNAi* | TCTAGAAGCTGCACAGATGCTCTC | GGATCCATGCCGCCCTTGAACTTG |
| *bc1*-CAPS | CAGGCGCTCATGACGTGGAC | GATCTTGGCGCGCCAGTAGTC |
| *bc11*-CAPS | CAGGTTCTCCGGTGGGCTCT | CATGGCGAAGAACACCTTGC |
| *cesa7-tos17* | TTCCTCAAGTTCCGGATCACGA | TCTTGCAGAAGGGGACCCAC |
| *Cesa9-tos17* | ATCGCCATCGTCATTGCTGATTCT | CCAAGCAAACCAGAAATTTCGATC |
| BC1 antigen | CAAGGACGGTGCGGCCACGACGGCTACC | ATGTCGACAGCCCCGTCCTTGCGCG |
| *BC1-RT* | TCGCCATCACAAACTTCAAC | TTTCGCATCAGCACCTCC |
| *UBQ5* | CCCTCCACCTCGTCCTCAG | AGATAACAACGGAAGCATAAAAGTC |
| *eEF1α* | TTTCACTCTTGGTGTGAAGCAGAT | GACTTCCTTCACGATTTCATCGTAA |
